# Frequency response compensation to optimize pretrained deep learning models in lung nodule malignancy classification

**DOI:** 10.3389/frai.2026.1934912

**Published:** 2026-09-22

**Authors:** V. Sangeetha, S. Kalaivani

**Affiliations:** School of Computer Science Engineering and Information Systems, Vellore Institute of Technology, Vellore, India

**Keywords:** Bode plot, deep learning, frequency response, Lag-Lead compensation, lung nodule, malignancy classification

## Abstract

**Purpose:**

Accurate differentiation of benign and malignant pulmonary nodules on computed tomography (CT) is important for early lung-cancer assessment. However, classification remains challenging because benign and malignant nodules may exhibit subtle differences in texture, margin irregularity, boundary sharpness, and internal morphology that may not be fully represented by conventional convolutional feature extraction.

**Methods:**

A frequency response Bilinear Lag-Lead (BT-LL) EfficientNetB3 framework was developed for benign-malignant pulmonary nodule classification. The proposed approach integrates Lag-Lead frequency compensation into the pretrained feature-extraction process to refine intermediate representations. The Lag component performs structural smoothing to preserve low-frequency information, whereas the Lead component enhances high-frequency boundary and texture characteristics. These corresponding representations are integrated with the original feature maps through a dedicated fusion operation to obtain frequency-refined features for classification. The compensation filters are fixed, thereby avoiding additional parameter learning within the filtering operation. Patient-level data partitioning was used to ensure that nodules from the same patient were not distributed across different experimental subsets, reducing the risk of information leakage.

**Results:**

The proposed model achieved an accuracy, sensitivity, and specificity of 0.90, an F1-score of 0.81, and an AUROC of 0.95 on the held-out patient-level test set. Performance was additionally assessed using patient-level cross-validation, cross imbalance analysis, an IQ-OTH/NCCD image-level evaluation and an independent BUS-BRA patient-level evaluation. The experimental analysis further included statistical significance testing using McNemar’s test for pairwise model comparisons, followed by the Holm correction to control for multiple comparisons, while Cochran’s Q test was employed to assess performance differences across multiple model configurations. The combined configuration achieved the best sensitivity and balanced accuracy relative to the branch-wise variants. The Bilinear Lag-Lead (BT-LL) framework thus provides an explicit, frequency response mechanism for integrating contributions of low- and high-frequency information in pulmonary nodule representations.

## 1 Introduction

Lung cancer remains one of the major causes of cancer-related mortality, and early diagnosis is essential for improving patient survival. Pulmonary nodules detected on computed tomography (CT) are important radiological findings in lung cancer screening, and accurate discrimination between benign and malignant nodules is a critical step in clinical decision-making. However, benign-malignant lung nodule classification remains challenging because nodules may show subtle differences in size, shape, density, margin irregularity, spiculation, calcification, and internal texture (Du et al., 2025).

CT-based lung nodule assessment is affected by several imaging-related factors, including small nodule size, low contrast between the lesion and surrounding lung tissue, blurred boundaries, vessel-adjacent structures, and heterogeneous nodule morphology. These factors increase the difficulty of both manual radiological interpretation and automated computer-aided diagnosis. Recent studies have therefore emphasized the need for robust artificial intelligence-based approaches that can extract discriminative imaging features from CT images for lung nodule and lung cancer analysis (Du et al., 2025). Deep learning has become an important approach for lung nodule malignancy classification because convolutional neural networks can automatically learn hierarchical features from medical images. Pretrained deep learning models are especially useful in medical imaging because annotated datasets are often limited in size. EfficientNet-based architectures have shown promising performance in CT-based lung cancer classification by providing an effective balance between classification accuracy and computational efficiency (Raza et al., 2023). However, standard pretrained convolutional networks are mainly data-driven and may not explicitly preserve frequency-specific information related to nodule structure and boundary characteristics.

Recent pulmonary nodule classification studies have explored attention-based architectures, self-supervised and multimodal learning, and neural architecture search to improve discriminative feature representation (Zheng et al., 2024; Cao et al., 2024; Sun et al., 2024; Yu et al., 2025); these approaches are reviewed in detail in Section 2.1. Despite these advances, there remains a need for explicitly frequency-aware feature-refinement strategies that can enhance combined structural and boundary information through a structured and interpretable mechanism.

In lung nodule malignancy classification, both low-frequency structural information and high-frequency boundary information are clinically meaningful. Low-frequency components preserve coarse nodule shape and structural continuity, whereas higher-frequency components emphasize margin irregularity and fine structural details. Encouraged by the frequency-selective behavior of classical Lag-Lead compensation (Lu et al., 2023), and used here as a frequency-response analogy rather than a literal control-loop implementation, the proposed framework introduces lag-like smoothing and lead-like detail enhancement to refine pretrained CT features.

Motivated by this gap, this work proposed a frequency response compensation strategy to customize pretrained deep learning models for lung nodule malignancy classification. The proposed approach integrates Lag-Lead compensation into the feature-extraction Compensation of a pretrained model to provide better feature refinement. The compensation stage is designed to balance structural smoothing and boundary enhancement while avoiding additional trainable parameters in the compensation filters. This provides a computationally efficient and interpretable feature-refinement mechanism compared with purely data-driven attention or fusion-based methods (Zheng et al., 2024; Cao et al., 2024; Sun et al., 2024; Yu et al., 2025).

The main contributions of this work are as follows. First, a frequency response compensation mechanism by Lag and Lead concepts is introduced for CT based benign-malignant pulmonary nodule classification. Second, spatial frequency filters are incorporated into a pretrained EfficientNetB3 framework and combined through learned feature fusion to refine intermediate representations using combined structural and boundary information. Third, the proposed framework is systematically evaluated through stage-wise and component ablations, parameter-sensitivity analysis, computational-complexity assessment, patient-level held-out testing, calibration analysis, and paired statistical comparisons. The results demonstrate the potential of frequency response feature refinement as a structured and interpretable strategy for medical image classification (Du et al., 2025; de Margerie-Mellon and Chassagnon, 2023; Raza et al., 2023; Lu et al., 2023). The method is evaluated under a leakage-conscious patient-level data-partitioning protocol using validation-based threshold selection, AUROC, average precision, calibration measures, confidence intervals, and exact paired statistical tests.

## 2 Related works

The differentiation of benign and malignant pulmonary nodules from computed tomography (CT) remains a difficult image-classification problem because clinically relevant differences may be manifested through relatively subtle variations in margin sharpness, local texture, internal heterogeneity, shape, and surrounding tissue structure. Deep convolutional neural networks (CNNs) have considerably improved automatic feature learning for this task, but conventional convolutional processing does not explicitly regulate how low- and high-spatial-frequency information is represented at different stages of the feature hierarchy. This issue is particularly relevant to pulmonary nodules because smooth intensity variations and broader structural context coexist with fine boundary irregularities and heterogeneous internal texture. Consequently, two related research directions have emerged: increasingly sophisticated deep-learning architectures for nodule malignancy prediction and explicit spatial-frequency or multi-resolution processing. The present study lies at the intersection of these directions by introducing Lag-Lead low and high-frequency compensation within a pretrained EfficientNetB3.

### 2.1 Deep learning for lung nodule malignancy classification

Transfer learning has become an important strategy for medical-image classification because it permits deep visual representations learned from large-scale image collections to be adapted when task-specific medical data are comparatively limited. EfficientNet is particularly relevant in this context because its compound-scaling strategy jointly balances network depth, width, and input resolution instead of scaling these dimensions independently (Tan and Le, 2019). The architecture demonstrated strong transfer capability with substantially fewer parameters than many earlier CNN families, providing a reasonable basis for developing computationally efficient medical classifiers. However, adopting a pretrained architecture alone does not ensure that diagnostically important fine-scale texture and boundary information is optimally preserved during feature extraction. The problem has therefore motivated modifications based on multi-scale representation, attention, auxiliary supervision, and multimodal information.

Zheng et al. (2024) addressed nodule heterogeneity through an attention-guided multichannel network that generated multi-scale and multi-view representations of pulmonary nodules. Three attention-based submodels extracted combined features before final prediction. On LIDC-IDRI, the method reported an accuracy of 90.11% and an AUC of 95.66%. The study demonstrates that directing the network toward scale- and view-dependent nodule characteristics can improve discrimination without requiring a conventional single-view representation. Its limitation relative to the present work is that the improvement is achieved by expanding the feature-extraction architecture through multiple image representations and attention branches; it does not directly investigate how the frequency response of intermediate CNN features influences malignancy discrimination.

More recent studies have attempted to overcome the ambiguity between morphologically similar benign and malignant nodules by incorporating information beyond image pixels. Sun et al. (2024) developed Nodule-CLIP, in which CT-derived image representations, malignancy categories, and radiologist-provided nodule attributes are aligned through multimodal contrastive learning. The method reported a benign/malignant classification accuracy of 90.6% and recall of 92.81% on LIDC-IDRI. This approach is important because it demonstrates that semantic attribute information can constrain the representation space and help discriminate nodules with similar visual appearances. Nevertheless, it depends on segmentation and additional semantic annotations and therefore addresses the problem primarily through richer supervision rather than through refinement of the intrinsic spatial-frequency response of a pretrained visual network (Imtiaz et al., 2023; Shah et al., 2024; Hasenstab et al., 2024; Guo et al., 2025; Hiraman et al., 2024).

Cao et al. (2024) approached the scarcity of labeled nodules through SSTL-FM, a self-supervised transfer-learning and fusion framework. Their strategy transfers representations learned from non-nodule CT regions and subsequently combines image features with clinical attributes; an additional feature-refinement module promotes interaction between the two feature sources. SSTL-FM therefore addresses two important problems limited labeled samples and incomplete image-only representations but requires auxiliary data, self-supervised pretraining, clinical attributes, and feature-fusion mechanisms. In contrast, the motivation of the present study is to determine whether a pretrained image classifier can itself be improved through a lightweight and deterministic modification of its internal feature response.

A related multimodal direction is represented by ETMO-NAS, proposed by Yu et al. (2025) for PET/CT lung-nodule classification. ETMO-NAS employs a two-step one-shot neural architecture search strategy to identify efficient multimodal feature extractors and reported accuracy, sensitivity, and specificity of 94.23, 95.83, and 92.86%, respectively. The selected architectures used substantially fewer parameters than several CNN and NAS comparators (Hammad et al., 2025; Tang et al., 2024; Naik et al., 2024; Liu et al., 2026; Xue et al., 2025). Although the work demonstrates that high diagnostic performance and architectural efficiency are not mutually exclusive, its PET/CT multimodal formulation and architecture-search procedure address a substantially different optimization problem from fixed feature-level frequency compensation in a pretrained CT classifier.

Investigations from the IQ-OTH/NCCD dataset itself also demonstrates that sophisticated feature fusion can produce high internal classification performance. Aaseegha and Venkataramana (2026) proposed a double-attention Hybrid CNN-HiFuse model incorporating hierarchical multi-scale feature fusion with channel and spatial attention. Their three-class model reported 98.12% test accuracy and 98.13% F1-score on a patient-level test split of IQ-OTH/NCCD, while the authors explicitly cautioned that the single-center dataset and very high performance require external validation. This result is important when interpreting the present work: high within-dataset performance on IQ-OTH/NCCD is achievable by several architectural strategies, and therefore classification accuracy alone cannot establish the novelty or superiority of the proposed Lag-Lead approach. The contribution must instead be justified by its mechanism, parameter efficiency, ablation behavior, and eventual generalization to independent patient cohorts.

Taken together, current lung-nodule classifiers increasingly improve representation through attention, multimodal fusion, self-supervision, architecture search, and hierarchical feature fusion. These approaches confirm that standard convolutional representations leave room for task-specific refinement. At the same time, most optimize where the model attends, which auxiliary information it learns from, or how multiple representations are fused. They do not directly constrain the spatial-frequency response of intermediate pretrained features. This distinction motivates examination of frequency-aware representations as a separate but contribution research direction.

### 2.2 Frequency-aware representation for pulmonary nodule classification

Frequency-aware processing is particularly relevant to image classification because feature maps contain information operating at different spatial scales. Low-frequency components primarily represent slowly varying spatial structure, whereas higher-frequency responses emphasize rapid local variation such as boundaries, fine texture, and small structural transitions. The usefulness of separating these components has been demonstrated by several general-purpose CNN architectures. Octave Convolution, for example, factorizes feature maps into high- and low-spatial-frequency groups processed at different resolutions; importantly, its objective is not to increase complexity but to reduce spatial redundancy and computational cost while preserving cross-frequency communication (Chen et al., 2019). Fast Fourier Convolution similarly combines local, semi-global, and global branches, using spectral processing to provide non-local receptive fields with complexity reported as comparable to ordinary convolution (Chi et al., 2020). FcaNet instead interprets global-average pooling as a special case of frequency decomposition and uses DCT-derived multi-spectral representations for channel attention, while retaining the same parameter count and computational cost as the corresponding SENet baseline (Qin et al., 2021). These studies establish the value of frequency-aware feature manipulation (Zhang et al., 2024) but differ fundamentally from the present approach because their objective is to redesign convolutional or attention operators rather than provide fixed residual compensation to an existing pretrained feature hierarchy.

Stuchi et al. (2024) more directly investigated spectral learning by constructing a frequency-domain image-classification architecture containing trainable Fourier-domain filters. Their framework learns discriminative spectral components and combines global and local frequency information while remaining comparatively lightweight; the experiments included texture classification as well as cataract and retinal-image tasks. The study provides strong proof that discriminative information can be explicitly extracted from image spectra instead of being left entirely to conventional spatial convolutions. However, its spectral filters are optimized through training and operate within a dedicated frequency-domain architecture. The present work asks a different question: whether useful frequency bias can be introduced without learning the frequency filters themselves, while retaining the pretrained EfficientNet feature extractor.

More importantly, explicit frequency processing has already been investigated directly for pulmonary nodule malignancy classification. Masquelin et al. (2021) evaluated discrete wavelet decomposition as an alternative to learned convolutional operations in a small-data setting using nodules from the National Lung Screening Trial. Their balanced dataset contained 354 malignant and 354 benign nodules, represented through axial, coronal, and sagittal views. Multi-level DWT achieved an AUC of around 94%, compared to around 92% for the conventional convolutional alternative, while reducing the number of learned parameters and speeding convergence. This study is very relevant in that it demonstrates that prespecified frequency filters can be beneficial for benign-malignant lung-nodule classification, particularly when the training data are limited. Its architecture however replaces early convolutional processing with wavelet decomposition and explicitly performs multi-resolution transformation. It does not work fixed combined low-pass/high-pass residual compensation within a pretrained MBConvolution hierarchy.

An even closer contemporary comparator is WaveLCDNet, introduced by Halder (2025). WaveLCDNet combines a conventional CNN mechanism with trainable wavelet blocks and CBAM attention to jointly model spatial and multi-resolution frequency features. The method achieved 96.70% accuracy, 96.89% sensitivity, and 95.52% specificity on LIDC-IDRI and was subsequently evaluated on an independent DSB2017 test set, where it reported 95.90% accuracy. This is particularly important for positioning the present research because it demonstrates that frequency-aware pulmonary-nodule classification is no longer an unexplored area. The research gap must therefore be stated more precisely. WaveLCDNet learns wavelet-related representations through a two-mechanism network and combines them with attention, whereas the proposed approach keeps the Gaussian and Laplacian coefficients fixed and introduces them directly within existing pretrained MBConvolution blocks. Thus, the intended contribution is not simply “using frequency information for lung-nodule classification,” but developing a substantially simpler feature-response compensation mechanism that modifies an already pretrained architecture without requiring frequency filters, explicit wavelet decomposition, or an additional attention mechanism.

Classical filter structures have also been integrated into deep networks in other medical applications. Cantone et al. (2023) proposed DoG-MCNet for mammographic microcalcification detection, placing a Difference-of-Gaussians layer ahead of the CNN. Rather than fixing the filters, the Gaussian standard deviations were optimized during training, allowing the network to learn a bank of band-pass filters. The resulting DoG-MCNet improved AUFROC by 4.86 percentage points relative to baseline MCNet and by 1.53 points relative to the reported multicontext CNN ensemble. DoG-MCNet is therefore an important methodological comparator: both approaches explicitly introduce conventional frequency-selective filtering into a medical deep-learning pipeline, but DoG-MCNet learns the filter bandwidth at the input/first layer, whereas the proposed framework uses fixed filters at intermediate pretrained feature levels.

Gabor-based CNNs provide a further example of structured filtering. Luan et al. (2018) incorporated Gabor functions into convolutional networks to improve robustness to orientation and scale changes while reducing the number of learned parameters. Their objective is principally directional and scale-selective representation rather than joint smoothing and detail compensation. Nevertheless, together with wavelet, DoG, Fourier, and DCT-based approaches, these studies demonstrate a consistent pattern: introducing prior knowledge about frequency or structured filtering can complement purely unconstrained learned convolution.

The Table 1 also illustrates why direct numerical ranking across studies would be inappropriate. The studies use different datasets, definitions of malignancy, input dimensionalities, patient/sample counts, preprocessing procedures, train/test partitions, and in some cases different imaging modalities.

**Table 1:** Critical comparison of representative studies relevant to frequency-response compensation for lung nodule classification.

Reference	Dataset	Frequency mechanism	Integration point	Frequency filters trainable?	Distinction
Masquelin et al. (2021)	NLST	DWT	Early representation	No	Explicit wavelet decomposition
Halder (2025)	LIDC-IDRI, DSB2017	Wavelet	Parallel mechanism	Yes	Trainable wavelet + CBAM
Stuchi et al. (2024)	Multiple	Fourier filters	Dedicated spectral network	Yes	Learned spectral filtering
Cantone et al. (2023)	Mammography	DoG	First/input layer	Yes	Learned Gaussian bandwidth
Chen et al. (2019)	ImageNet etc.	High/low frequency	Replaces convolution	Yes	Multi-resolution convolution
Qin et al. (2021)	ImageNet etc.	DCT	Channel attention	Fixed bases + learned attention	Frequency attention
Proposed	LIDC-IDRI/IQ-OTH/BUS-BRA	Gaussian + Laplacian+Lag-Lead	After depthwise Convolution, before Squeeze Excitation block	Fusion learned	Internal frequency-response compensation

### 2.3 Critical comparison with the proposed Lag-Lead compensation

The literature shows that frequency-based methods span a range of designs, rather than falling into a strict division between complex learnable modules and filtering approaches. Some frequency-aware architectures, such as OctConv, explicitly reduce computation, while FFC maintains complexity comparable to vanilla convolution and FcaNet can retain the same parameter count as its attention baseline. A more defensible distinction concerns what is learned, where the frequency operation is introduced, and whether the pretrained model must be structurally replaced or supplemented.

The proposed framework modifies EfficientNetB3 Mobile Inverted Bottleneck Convolution processing by inserting frequency-response compensation after depthwise convolution and before squeeze-and-excitation recalibration. This position is conceptually important because the depthwise convolution has already generated local spatial responses, while SE has not yet assigned channel-wise importance. The compensation module therefore operates on intermediate spatial features before channel recalibration rather than enhancing the raw CT image or replacing the convolution itself. The original feature map is accompanied by a Gaussian low-pass branch and a Laplacian high-pass branch, after which the combined representations are combined using a lightweight trainable fusion operation.

The Gaussian branch is interpreted as a lag component because it suppresses rapid spatial variation and emphasizes slowly varying structure, whereas the Laplacian branch is interpreted as a lead component because it emphasizes rapid transitions associated with boundaries and local texture. However, this terminology should be treated as a control frequency response interpretation rather than an assertion that the spatial kernels are mathematically identical to classical continuous- or discrete-time lag and lead compensators. This distinction is important because the novelty of the method ultimately rests on the architecture and experimentally demonstrated compensation behavior, not on terminology alone.

The proposed design also differs from learned spectral filters in a specific way: the Gaussian and Laplacian coefficients are fixed and receive no gradient updates. This does not mean that the complete compensation module has zero additional parameters, because the subsequent feature-fusion operation is learned. The defensible claim is therefore that the method introduces no trainable frequency-filter coefficients, limiting optimization to the lightweight fusion stage. This preserves deterministic smoothing and edge-enhancement behavior while allowing the network to adapt the relative contribution of the original, low-frequency, and high-frequency features.

Despite substantial progress in attention-based, multi-scale, wavelet, Fourier, DoG, and frequency-channel methods, several limitations remain. Existing approaches commonly rely on trainable spectral filters, explicit multi-resolution decomposition, additional attention mechanisms, replacement of conventional convolutional operators, or increased architectural complexity. Moreover, relatively few studies investigate whether deterministic low- and high-frequency feature responses can be introduced directly within intermediate layers of a pretrained CNN while preserving the original structure. The central challenge is therefore to enhance combined structural and detail-sensitive information without substantially increasing the number of trainable parameters or disrupting pretrained representations. This motivates the proposed framework, which introduces fixed Gaussian low-pass and Laplacian high-pass responses at selected EfficientNetB3 feature stages and employs lightweight learned fusion to integrate the original and compensated representations.

## 3 Materials and methods

### 3.1 Dataset

The proposed framework was validated with three different datasets. The main dataset for model development and evaluation was the LIDC-IDRI dataset, was used to evaluate the cross-domain generalization ability of the proposed method.

#### 3.1.1 LIDC-IDRI dataset data partitioning

The Lung Image Database Consortium and Image Database Resource Initiative (LIDC-IDRI) dataset is a publicly available thoracic computed tomography (CT slice) database widely used for the development and evaluation of computer-aided lung nodule detection and classification systems. The dataset was established through a collaborative effort involving seven academic institutions and eight medical imaging companies and is publicly distributed through The Cancer Imaging Archive (TCIA). The complete LIDC-IDRI collection contains 1,018 thoracic CT slice cases, with each case accompanied by an XML annotation file containing lesion assessments provided by experienced thoracic radiologists.

Each CT examination was evaluated by up to four experienced thoracic radiologists using a two-phase annotation protocol consisting of blinded and unblinded reading stages. During the initial blinded phase, the radiologists independently identified pulmonary abnormalities, while in the subsequent unblinded phase they reviewed their own annotations together with anonymized annotations from the other radiologists before providing their final assessments. Pulmonary findings were categorized primarily as nodules measuring at least 3 mm, nodules smaller than 3 mm, or non-nodule abnormalities. For nodules measuring at least 3 mm, detailed contour annotations and subjective radiological characteristics, including subtlety, margin, spiculation, lobulation, sphericity, texture, and likelihood of malignancy, are provided.

For the present work as shown in Table 2, the original CT Slices and corresponding radiologist annotations were processed to construct a binary lung nodule classification dataset. Following the predefined inclusion and labeling criteria, 1,073 slice eligible nodule images from 581 unique patients were retained. Among these images, 781 were categorized as benign slices and 292 as malignant slices. Patient identifiers were preserved throughout preprocessing to enable patient-level data partitioning and to ensure that images originating from the same patient were not distributed across different training, validation, or test subsets. This patient-disjoint strategy reduces the possibility of information leakage and provides a more reliable assessment of the models ability to generalize to previously unseen patients (Varoquaux and Cheplygina, 2022).

**Table 2:** Original LIDC dataset and experimental subset used in this work.

Dataset stage/subset	Slices	Patients	Benign slices	Malignant	Indeterminate	Ambiguous/unmatched
Samples identified during preprocessing	2,630	–	781	292	1,550	7
Excluded from analysis	1,557	–	0	0	1,550	7
Final analytic cohort	1,073	581	781	292	0	0
Training set	757	416	549	208	–	–
Validation set	152	80	108	44	–	–
Test set	164	85	124	40	–	–
Total patient-level partition	1,073	581	781	292	–	–

The patient identifier of each slices was extracted and used to split the data to avoid leakage at the patient level. Thus, all images of a same patient were included in only one data subset. A patient-disjoint stratified splitting strategy was employed to maintain an approximately representative class distribution in training, validation, and test subsets, while ensuring that no single patient is present in more than one subset. We proposed a more robust evaluation protocol for model generalization, where images from the same patient are not correlated between the training and evaluation sets.^1^

### 3.2 Image standardization

A standardized preprocessing pipeline was used to make sure that the results were consistent and compatible with deep learning architectures.

#### 3.2.1 Spatial standardization

All normalized images were resized to 300 × 300 pixels using bicubic interpolation to match the EfficientNetB3 input size as shown in Equation 1:


$$I_{\text{resized}}={\text{Resize}}_{\text{bicubic}}(I_{\mathrm{norm}},300\times 300)$$ (1)

To retain high-frequency edge data, which is important in the detection of nodule boundaries, bicubic interpolation was chosen. The resulting image *I_resized_* serves as the input to the proposed work.

### 3.3 Why compensation?

Conventional Lag-Lead compensators are designed to shape the frequency response of a system by selectively modifying the contribution of different frequency components. In this work, this principle is adopted as a frequency-response analogy rather than a literal implementation of a control loop. The objective is to balance information from different frequency responses in CNN feature maps. The Lag component emphasizes low-frequency structural content while the Lead component emphasizes higher-frequency boundary and texture information. The proposed compensation mechanism is integrated with the original feature representation, which preserves the coarse nodule morphology and improves the diagnostically relevant details like margin irregularity and spiculation.

#### 3.3.1 Frequency-response compensation module

The proposed module introduces combined low-frequency preservation and edge-sensitive feature modulation into intermediate CNN feature maps. The terminology lag and lead is used as a control interpretation of the frequency-response shaping. The implementation should not be interpreted as applying a continuous-time controller directly to an image. Instead, continuous first-order prototypes are discretized using the bilinear transformation and implemented as spatial first-order recurrences. These recurrences operate after fixed Gaussian and Laplacian depthwise spatial prefilters.

The fixed spatial kernels and the recursive sections have distinct functions. The Gaussian and Laplacian kernels determine the basic low-pass and high-pass spatial priors, respectively, whereas and modify the subsequent recursive response. The parameters used in the reported implementation were α = 0.2, *β* = 5.0, K = 1.0, *Ts* = 1.0, λ = 1.0 The selected operating point was *T1* = 0.2, *T2* = 0.1. These values correspond directly to the implemented configuration.

The Lag compensator acts as a low-pass filter as represents in Equation 2:


$$G_{\mathrm{lag}}(s)=K_{\mathrm{lag}}\frac{1+T_{1}s}{1+\beta T_{1}s},\beta >1$$ (2)

$G_{\mathrm{lag}}(s)$ : Transfer function of the Lag compensator, s=σ+jω : Complex frequency variable, ω : Angular frequency (spatial frequency in images), $K_{\mathrm{lag}}$ : Gain factor (controls overall intensity scaling), $T_{1}$ : Time constant (controls cutoff frequency), β : Attenuation factor ( β>1 ) controlling high-frequency suppression.

The continuous prototype is converted to a discrete first-order transfer function using the bilinear (Tustin) substitution (Cao et al., 2024):


$$s=\frac{2}{T_{s}}\frac{1-z^{-1}}{1+z^{-1}}$$ (3)

Substituting Equation 2 into Equation 3 and multiplying numerator and denominator by Ts(1 + z^−1^) gives Equations 4, 5:


$$H_{\mathrm{Lag}}(z)=\frac{b_{o}l+b_{1}Lz^{-1}}{a_{o}l+a_{1}Lz^{-1}}$$ (4)

$$\begin{array}{l} b_{o}L=K(T_{s}+2\beta T_{1}),b_{1}L=K(T_{s}-2\beta T_{1}),\\ a_{o}L=T_{s}+2T_{1},a_{1}L=T_{s}-2T_{1}) \end{array}$$ (5)

The corresponding spatial recurrence for a one-dimensional sequence x[n] is as shown in Equation 6:

$$y[n]=\frac{\left\{b_{0}x[n]+b_{1}x[n-1]-a_{1}y[n-1]\right\}}{a_{0}}$$ (6)

The code checks the discrete pole *p* = − $a_{1}$ / $a_{o}$ and rejects an unstable setting if |p| ≥ 1. With the reported parameter grid, all lag-path poles remain inside the unit circle.

In the implemented model, the desired low-pass behavior was realized using a fixed, non-trainable 3×3 spatial smoothing kernel applied independently to each feature channel. For an intermediate feature map X , the lag response was computed as using Equation 7:

$$X_{\mathrm{Lag}}=X*H_{\mathrm{Lag}}$$ (7)

Where $H_{\mathrm{lag}}$ denotes the fixed low-pass kernel and ∗ represents depthwise spatial convolution. The lag branch was intended to stabilize local feature variations without replacing the original feature representation.

#### 3.3.2 Lead compensation (high-frequency enhancement)

The Lead compensator enhances mid-to-high frequency components as presents in Equation 8:

$$G_{\text{lead}}(s)=K_{\text{lead}}\frac{1+T_{2}s}{1+\alpha T_{2}s},\alpha <1$$ (8)

This improves edge sharpness and enhances nodule boundary features critical for classification.

$G_{\text{lead}}(s)$ : Lead compensator, $K_{\text{lead}}$ : Gain parameter, $T_{2}$ : Time constant, α : Boost factor ( α<1 ), s : Frequency variable. In the implemented network, this behavior was represented using a fixed, non-trainable Laplacian-type spatial kernel shown in Equation 8.

The present implementation uses the following first-order transfer function in the second branch:

$$G_{\text{lead}}(s)=\frac{K(1-T_{2}S)}{\left(1+\alpha T_{2}S\right)}$$ (9)

Applying the same Tustin substitution yields in Equations 9, 10:

$$H_{\text{Lead}}(z)=\frac{b_{o}E+b_{1}Ez^{-1}}{a_{o}E+a_{1}Ez^{-1}}$$ (10)

$$\begin{array}{l} b_{o}E=K(T_{s}+2T_{2}),b_{1}E=K(T_{s}-2T_{2}),\\ a_{o}E=T_{s}+2\beta T_{2},a_{1}E=T_{s}-2\beta T_{2}) \end{array}$$ (11)

Then the same first-order recurrence of Equation 11 is used with the edge-branch coefficients. In the configuration used *β* = 5.0. Therefore, Equation 12 cannot be considered an independent classical high-frequency boosting lead compensator. The branch becomes edge enhancing since the input is first filtered by a fixed Laplacian high pass spatial pre-filter and the resulting recursively shaped edge response is then residually added to the identity representation. The new terminology “lead/edge-shaping branch” thus matches the actual computation and avoids overemphasizing the role of the continuous transfer function. For the selected values T2 = 0.1, *β* = 5.0, K = 1.0 and Ts = 1.0 and (b0, E, b1, E, a0, E, a1, E) = (1.20, 0.80, 2.00, 0.00) and therefore *p*_E_ = 0.

$$X_{\text{lead}}=X*H_{\text{lead}}$$ (12)

The lead response was then combined with the original feature map to enhance edge-sensitive information shown in Equation 13:

$${\tilde{X}}_{\text{lead}}=X+\lambda X_{\text{lead}}$$ (13)

Where λ=1.0 denotes the lead-enhancement strength. The 1 × 1 convolution is trainable and learns the relative importance of the identity, smoothed/lag-shaped and edge-enhanced representations.

#### 3.3.3 Extension of the 1D discrete filter to 2D feature maps

The present implementation does not perform the mapping by creating a 3 × 3 FIR kernel that is T-dependent. Instead, the first-order recurrence is applied directly on both spatial axis of each feature channel.

For a feature tensor *X ∈ ℝ^(H × W × C),* let *Fa(X) and Ba(X)* denote the forward and reverse first-order recurrences along spatial axis a ∈ {h,w}. Bidirectional filtering is obtained as in Equation 14

$$\mathrm{Ra}(X)=\frac{1}{2}[\mathrm{Fa}(X)+\mathrm{Ba}(X)]$$ (14)

and the two orthogonal responses are averaged to obtain the two-dimensional response utilizing Equation 15

$$R_{2}D(X)=\frac{1}{2}[\mathrm{Rh}(X)+\mathrm{Rw}(X)]$$ (15)

This allows extending the 1D transfer function to 2D with orthogonal axis-wise bidirectional recurrences and subsequent axis averaging. This operation is isotropy-oriented in the sense that height and width are treated symmetrically, but it is not mathematically identical to a radial 2D IIR or to a separable outer-product FIR. It is important to state this distinction explicitly for reproducibility.

#### 3.3.4 Gaussian and Laplacian filters

The feature map is smoothed with a depthwise Gaussian kernel prior to the T1-dependent lag recurrence. Before the T2-dependent edge recurrence, the feature map is convolved with a fixed depthwise Laplacian-type kernel. The exact deployed matrices are shown from Equations 16–19:

$$K_{G}=\frac{1}{16}[\begin{array}{ccc} 1 & 2 & 1\\ 2 & 4 & 2\\ 1 & 2 & 1 \end{array}]$$ (16)

$$K_{L}=\frac{1}{8}[\begin{array}{ccc} -1 & -1 & -1\\ -1 & 8 & -1\\ -1 & -1 & -1 \end{array}]$$ (17)

$$K_{G}=[\begin{array}{ccc} 0.0625 & 0.1250 & 0.0625\\ 0.1250 & 0.2500 & 0.1250\\ 0.0625 & 0.1250 & 0.0625 \end{array}]$$ (18)

$$K_{L}=[\begin{array}{ccc} -0.1250 & -0.1250 & -0.1250\\ -0.1250 & 1.000 & -0.1250\\ -0.1250 & -0.1250 & -0.1250 \end{array}]$$ (19)

Each matrix is repeated across channels and applied by depthwise convolution, so the filters do not introduce any trainable spatial coefficients.

#### 3.3.5 Lag lead fusion

Let denote the original feature map, the Lag-compensated representation, and the Lead-enhanced representation. These three feature maps are concatenated along the channel dimension and projected back to the original channel dimension using a bias-free convolution, followed by batch normalization and nonlinear activation is shown in Equation 20:

$$X_{\text{fusion}}=\sigma (\mathrm{BN}(W_{1\times 1}.\text{Concat}(X,X_{\mathrm{lag}},{\tilde{X}}_{\text{lead}})$$ (20)

The convolution parameters are learned independently at each compensation stage (S1–S4), enabling stage-specific integration of the original, low-frequency, and high-frequency feature representations. The combined design aimed to balance smoothing and boundary enhancement before the squeeze-and-excitation stage of the modified MBConvolution block.

#### 3.3.6 Effect of T1 and T2

T1∈{0.1, 0.2, 0.3}, T2∈{0.1, 0.2, 0.3} with α = 0.2, *β* = 5, K = 1, Ts = 1. The resulting coefficients are shown in Table 3.

**Table 3:** Lag and lead filter coefficients for different and parameter combinations.

(T1)	(T2)	Lag (b0)	Lag (b1)	Lag (a0)	Lag (a1)	Lag pole	Edge (b0)	Edge (b1)	Edge (a0)	Edge (a1)
0.1	0.1	1.04	0.96	1.20	0.80	−0.6667	1.20	0.80	2.00	0.00
0.1	0.2	1.04	0.96	1.20	0.80	−0.6667	1.40	0.60	3.00	−1.00
0.1	0.3	1.04	0.96	1.20	0.80	−0.6667	1.60	0.40	4.00	−2.00
0.2	0.1	**1.08**	**0.92**	**1.40**	**0.60**	**−0.4286**	**1.20**	**0.80**	**2.00**	**0.00**
0.2	0.2	1.08	0.92	1.40	0.60	−0.4286	1.40	0.60	3.00	−1.00
0.2	0.3	1.08	0.92	1.40	0.60	−0.4286	1.60	0.40	4.00	−2.00
0.3	0.1	1.12	0.88	1.60	0.40	−0.2500	1.20	0.80	2.00	0.00
0.3	0.2	1.12	0.88	1.60	0.40	−0.2500	1.40	0.60	3.00	−1.00
0.3	0.3	1.12	0.88	1.60	0.40	−0.2500	1.60	0.40	4.00	−2.00

Bold values represents the T1 and T2 combination yield best value comparing with other combinations.

The Gaussian and Laplacian matrices are fixed for all configurations. The bilinear transformation instead determines the coefficients of the first-order spatial recurrence that follows each fixed prefilter. We have therefore provided the two exact fixed spatial kernels and, for all nine combinations, the corresponding recursive coefficients and pole locations. This distinction makes explicit where T1 and T2 enter the implemented computation.

Frequency-response analysis was performed at three levels: (i) the continuous prototype evaluated on the Tustin frequency grid, (ii) the corresponding one-dimensional discrete recursive response, and (iii) the actual two-dimensional composite response formed by the fixed spatial filter and height/width recurrence. The latter represents the response most closely corresponding to the deployed network operation.

#### 3.3.7 EfficientNetB3 background

EfficientNetB3 was selected as the architecture for several interlocking reasons. First, the EfficientNet family achieves its accuracy-to-computation ratio through compound scaling, in which network depth, width, and input resolution are scaled jointly according to a fixed coefficient rather than adjusted independently, and this balanced scaling has repeatedly been shown to outperform architectures scaled along a single dimension when computational budgets are constrained a relevant consideration given the moderate size of the CT and BUS-BRA datasets available for this work. Second, among the EfficientNet variants, B3 sits at a point in the scaling curve that offers meaningfully greater representational capacity than the smaller B0–B2 variants while remaining substantially lighter than B4 and above, making it a practical choice for a dataset regime where deeper or wider networks would be prone to overfitting. Third, EfficientNetB3 is widely pretrained on ImageNet, and its transferred low- and mid-level filters edge, texture, and blob detectors have been shown in the medical imaging literature to transfer reasonably well to grayscale radiological modalities such as CT and ultrasound images, despite the domain gap between natural and medical images. Finally, the MBConvolution (Mobile Inverted Bottleneck Convolution) block that underlies every stage of EfficientNetB3 provided a structurally convenient insertion point for the proposed compensator: because each MBConvolution block already separates expansion, depth wise filtering, and projection into discrete sub-stages, a frequency-domain compensation branch could be embedded immediately after the depth wise convolution without disturbing the blocks residual connectivity or its squeeze-and-excitation recalibration step. In short, EfficientNetB3 was not chosen merely for its benchmark performance, but because its internal structure offered a natural and minimally invasive site at which a Lag-Lead compensator an idea imported from classical control theory could be inserted without redesigning the architecture from scratch.

### 3.4 Proposed method

The proposed model shown in Figure 1, denoted Bilinear Lag-Lead EfficientNetB3, accepts CT slices images as separate but architecturally identical input streams. Each input image is resized and normalized to a fixed spatial resolution of 300 × 300 × 3 prior to entering the network, a resolution chosen to match the native input expectation of the EfficientNetB3 while preserving sufficient spatial detail for nodule-scale features to survive early downsampling. The normalized image is passed through a stem convolutional layer, which performs the initial channel expansion and coarse spatial reduction characteristic of EfficientNet-style architectures, before entering the four-stage MBConvolution. Each of the four stages contains a stack of MBConvolution blocks of increasing depth and channel width, following the standard EfficientNetB3 stage configuration, but with one structural departure from the canonical design: every MBConvolution block across all four stages embeds the Lag-Lead compensator, so that frequency-domain compensation is applied repeatedly and at multiple spatial resolutions as the feature maps progress through the network, rather than being applied once at the input or only at the final feature map.

**Figure 1:**
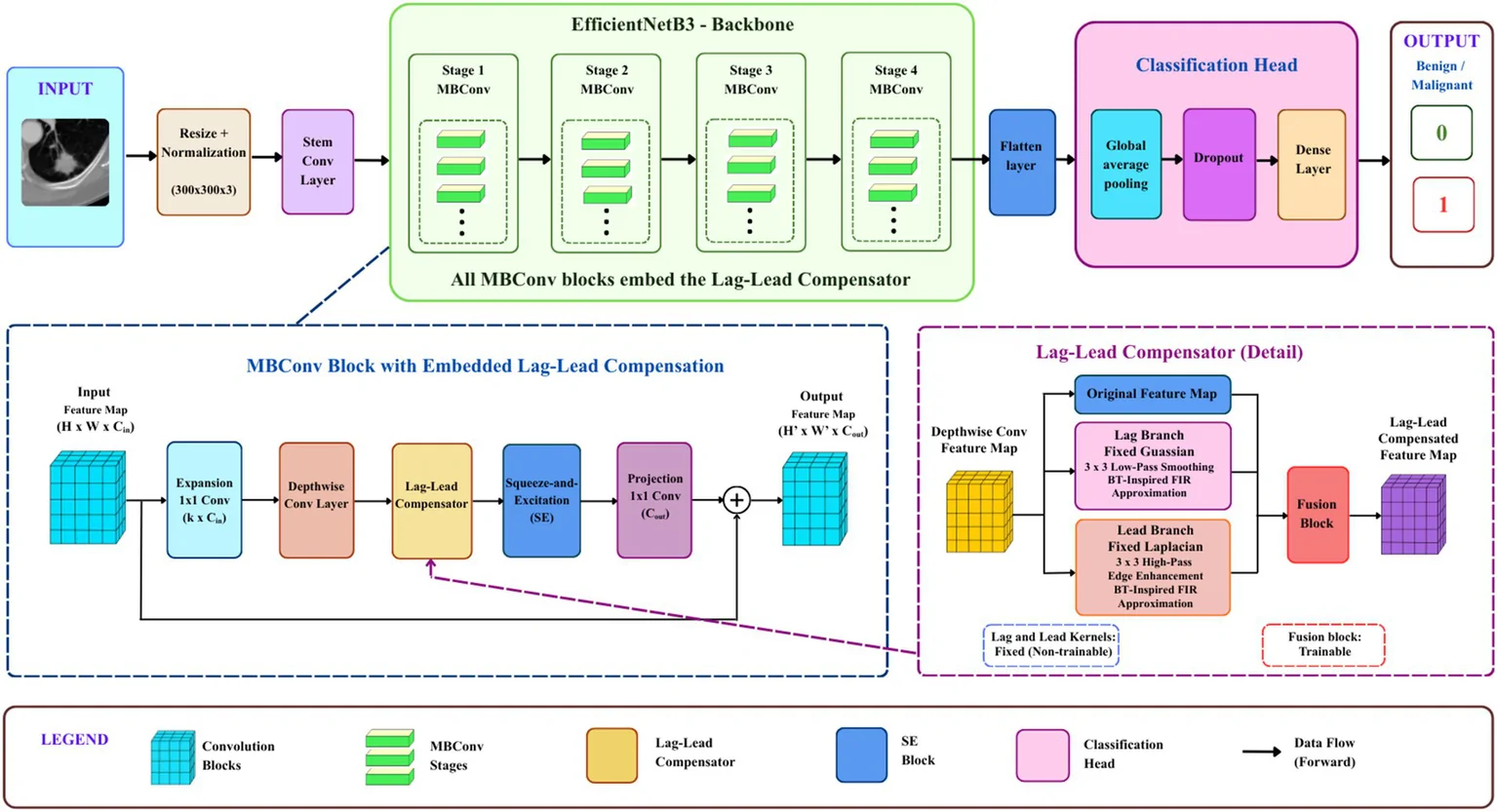
Proposed architecture.

Following the fourth stage, the resulting feature maps are flattened and passed to a lightweight classification head consisting of global average pooling, a dropout layer for regularization, and a final dense layer that produces the binary benign-versus-malignant prediction. This head is deliberately kept simple, since the discriminative burden in this architecture is intended to fall primarily on the network and its embedded compensation mechanism rather than on a heavily parameterized classifier.

#### 3.4.1 The proposed architecture

The internal structure of each modified MBConvolution block follows the standard expansion depth wise-projection pattern, with the Lag-Lead compensator inserted as an additional processing stage immediately after the depth wise convolutional layer and immediately before the squeeze-and-excitation block. This placement was chosen so that the compensator operates directly on the spatially filtered depth wise feature map the representation most analogous, in a loose signal-processing sense, to a filtered one-dimensional signal before that representation is recalibrated channel-wise by the squeeze-and-excitation mechanism and projected back down by the final 1 × 1 convolution. A residual connection carries the original block input forward to the output summation, preserving the standard MBConvolution mechanism and ensuring that the compensator behaves as an additive refinement rather than a replacement of the networks native feature transformation.

Within the compensator, the depthwise-convolution feature map is processed through three parallel representations: an identity path, a Lag path, and a Lead/edge-shaping path. The identity path preserves the original feature map. We distinguish the fixed spatial filtering operations from the $T_{1},T_{2}$ dependent on recursive operations. In the implemented BT-LL module, the 3 × 3 Gaussian and Laplacian depthwise kernels are fixed and non-trainable. Their coefficients remain unchanged across all $T_{1}$ and $T_{2}$ settings. The Gaussian kernel provides the low-frequency spatial prefilter for the Lag branch, whereas the Laplacian-type kernel provides the high-frequency/edge prefilter for the Lead branch.

These spatial kernels are not altered by $T_{1}$ and $T_{2}$ . Instead, after the corresponding fixed filter, the feature map is processed by a first-order bidirectional spatial recurrence. $T_{1}$ determines the Lag-branch recurrence coefficients and pole, whereas $T_{2}\;$ determines the corresponding coefficients of the Lead/edge-shaping recurrence. The values of K , α , β , and $T_{s}$ are held fixed in the reported implementation. Table 3 now explicitly reports the recurrence coefficients for all evaluated $T_{1}\;\text{and}\;T_{2}$ combinations while keeping the Gaussian and Laplacian kernels unchanged.

Both the lag and lead kernels remain common throughout training, in keeping with the intended design in which the compensation filters encode a prior structural assumption about smoothing and edge-sharpening rather than being left free to drift during optimization. The outputs of the identity, lag, and lead paths are then combined in a fusion block, which unlike the compensation kernels themselves is fully learnable, allowing the network to learn the optimal weighting between the unfiltered, smoothed, and edge-enhanced representations at each depth of the architecture. The remaining compensator parameters, namely the static gain K = 1.0, the compensator ratio *β* = 5.0, the pole-zero spacing factor α = 0.2, and the sampling interval Ts = 1.0, were held constant across all stages and are reported here as common reference values used throughout the bilinear-transform derivation that motivated the FIR kernel design.

It is worth noting explicitly, for methodological transparency, that the lag and lead kernels implemented in the network are spatial FIR filters rather than the continuous-time transfer function from which they were derived; the bilinear-transform derivation serves as the theoretical justification and parameter source for the kernel coefficients, but the deployed compensator operates as a discrete convolutional filter bank, and this distinction is maintained consistently between the mathematical formulation and the implemented architecture described above.

## 4 Experimental results

### 4.1 Model evaluation

The proposed work was evaluated using dataset-specific protocols. LIDC-IDRI served as the primary development and patient-level evaluation dataset. IQ-OTH/NCCD was evaluated using stratified image-level folds because reliable patient identifiers were unavailable in the public release, and its results are therefore treated as exploratory. Training, validation and testing subsets were created by dividing samples available for each dataset with a ratio of 70:15:15. The splitting was done at the patient level to avoid information leakage between the subsets. The training subset was used to optimize the model, the validation subset to monitor the model, select the operating threshold and evaluate the parameters. The held-out test subset remained independent of model progress and was used only for the final evaluation of the selected model.

To make a fair comparison, all configurations of the models were trained for 60 epochs with the same optimization and preprocessing settings. The compensation gain was kept constant at K = 1 and the selected lag and lead parameters were T1 = 0.20 and T2 = 0.10, respectively. The experiments were performed by separately training the baseline model of EfficientNetB3, lag-only compensation, lead-only compensation and the combined Lag-Lead compensation model. In the proposed configuration, the compensation module was added to the selected EfficientNetB3 feature stages according to the experimental design described in the previous sections.

The model performance was evaluated by the confusion-matrix components of true positives (TP), true negatives (TN), false positives (FP) and false negatives (FN). Accuracy, balanced accuracy, sensitivity (recall), specificity, precision, negative predictive value (NPV), F1-score, and Matthews correlation coefficient (MCC) were the values obtained. To provide a more informative measure, in cases of class imbalance, balanced accuracy and MCC were added.

AUROC (area under the receiver operating characteristic curve) and AP (average precision) were used to assess threshold-independent discrimination. The AUROC quantifies the model’s overall performance in discriminating between malignant and benign nodules at the decision thresholds, while the AP provides an additional measure that is particularly informative when the class distribution is imbalanced.

Apart from discrimination performance, the probabilistic reliability was evaluated by the Brier score and the expected calibration error (ECE). The Brier score measures the mean squared error between the predicted probabilities and the true outcomes. The ECE measures the expected calibration error, which is the agreement between the confidence of the prediction and the actually observed class frequency. Lower values of both metrics are better calibrated probability estimates.

Confidence intervals were estimated for the main performance measures where appropriate to quantify the uncertainty associated with the held-out test results. Statistical comparisons between models were made using paired tests on the same test samples. Cochran’s Q test was used as an omnibus test when more than two related model configurations were compared, followed by exact McNemar tests for relevant pairwise comparisons with correction for multiple testing. This evaluation protocol allowed the assessment of both the predictive performance and the statistical robustness of the proposed Lag-Lead compensation framework across the two datasets.

### 4.2 Evaluation implications

We assessed the model and computed four important metrics: recall, precision, test accuracy, and F1 score. The precision, recall, F1 score, and accuracy equations are given by Equations 21–24, respectively.

The percentage of all samples that are correctly predicted to be either positive or negative is known as test accuracy. It is calculated using the Equation 21:

$$\text{Accuracy}=\frac{\mathrm{TP}+\mathrm{TN}}{\mathrm{TP}+\mathrm{TN}+\mathrm{FP}+\mathrm{FN}}$$ (21)

TP = True positive; TN = True negative; FP = False positive; FN = False negative.

True positive (TP): This indicates that the model was successful in identifying a positive example because both the actual and estimated data are good.
False Positive (FP): The actual value is negative while the expected value is positive. A positive and a negative were combined in the model.
True Negative (TN): The model has chosen a negative value if both the predicted and actual values are negative.
False Negative (FN): The actual value is positive, whereas the expected value is negative. This indicates that a positive case was incorrectly identified as negative by the model.

The precision indicator is calculated by dividing the number of correctly predicted positive samples by the total number of anticipated positive samples. It is a useful indicator of the models effectiveness and performance as well as a crucial indicator of how well the models recognize positive cases. This is calculated using Equation 22:

$$\text{Precision}=\frac{\mathrm{TP}}{\mathrm{TP}+\mathrm{FP}}$$ (22)

Recall, also known as sensitivity or the valid positive rate, is a performance metric that assesses a models accuracy in identifying positive samples. The Equation 23 used to determine recall is:

$$\text{Recall}=\frac{\mathrm{TP}}{\mathrm{TP}+\mathrm{FN}}$$ (23)

As indicated by Equation 24, the F1-score is a statistic that determines the precision and recall of a binary classification model. There is a method for calculating this:

$$F1\;\text{score}=\frac{2\;X\;\text{Precision}\;X\;\text{recall}}{\text{Precision}+\text{Recall}}$$ (24)

### 4.3 Experimental setup and classification performance

The developed proposed work used Python 3.12.13, Tensorflow 2.20.0, Keras 3.3.3 and CUDA 13.0. Preprocessing and initial code development was done on 13th Gen Intel(R) Core(TM) i5-1335U processor with 64-bit Windows system with 13th Gen Intel(R) Core(TM) i5-1335U processor and NVIDIA GeForce RTX 3050 U Laptop GPU. The computationally demanding model training and evaluation was done on Google Colab Pro with NVIDIA A100 GPU with 40 GB VRAM, Cuda 13.0. Additional experiments were conducted in Kaggle notebooks with NVIDIA Tesla P100-PCIE-16GB GPU (16 GB VRAM, compute capability 6.0). All CT images Slices were resized to (300 × 300) pixels, to satisfy the input-size requirements of EfficientNetB3 architecture. The model was trained for 60 epochs with batch size 8 for all the Experiments. Figure 2 shows the Elastic deformation (Wang Z. et al., 2025) was used as a training-time augmentation strategy to simulate small non-rigid variations in pulmonary nodule appearance. Smooth displacement fields were applied to the CT images to perturb local shape and boundary geometry while retaining the underlying benign or malignant label. The augmentation was restricted to the training data after patient-level partitioning, whereas validation and test sets were evaluated without augmentation.

**Figure 2:**
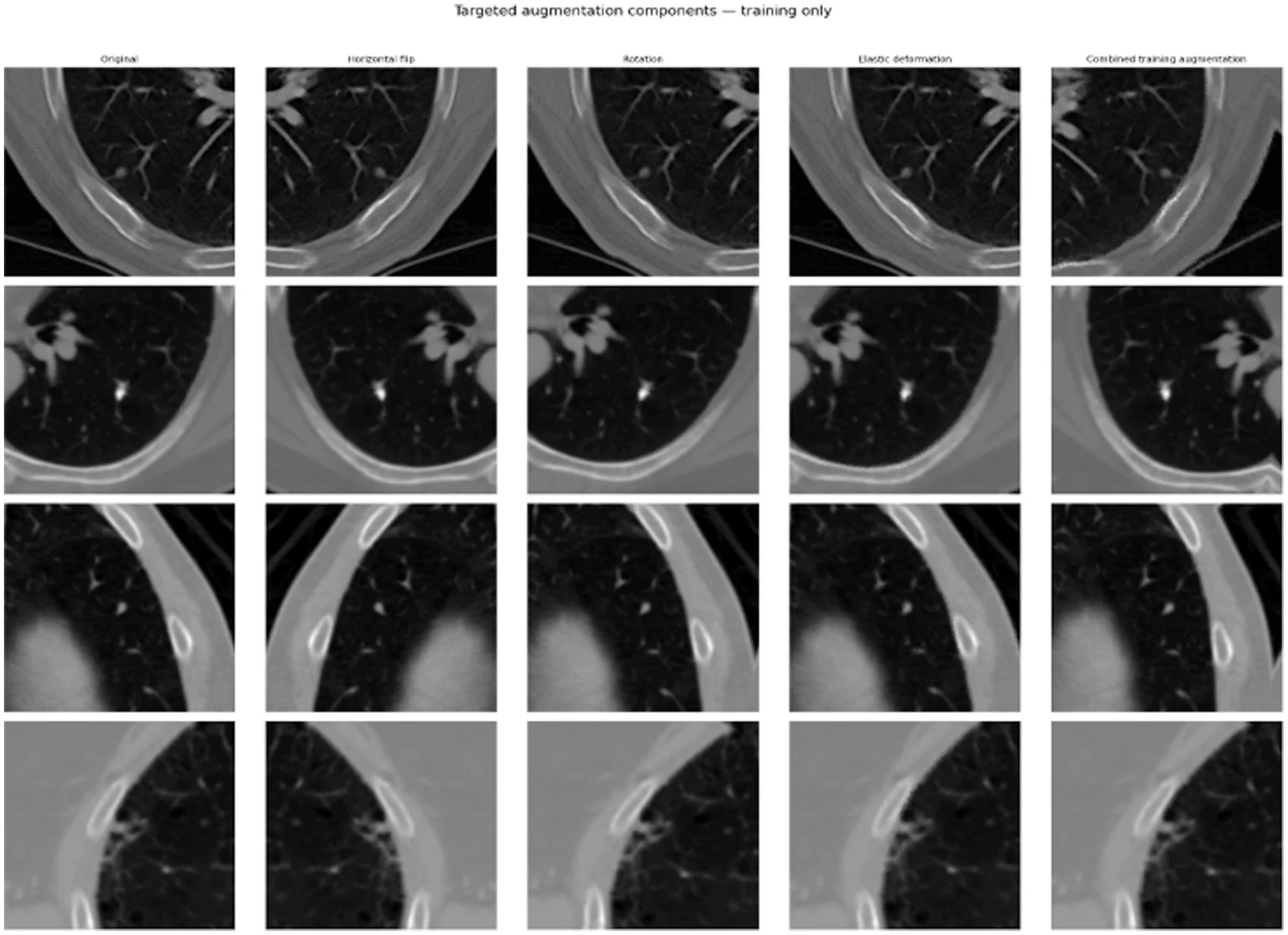
Representative Lung nodule CT images used for training data augmentation.

We used the Adam optimizer with a fixed learning rate (1 × 10^−4^). In this work, the binary classification of benign and malignant lung nodules was used, so the loss function was binary cross-entropy. To reduce overfitting, a dropout rate of 0.3 was used on the fully connected classification layer to randomly disable a proportion of neurons during training. The output layer was the last layer and it used sigmoid activation function to output a probability value between 0 and 1. Samples whose predicted probabilities were larger than or equal to the chosen classification threshold were classified as malignant and the remaining samples were classified as benign.

The test-set classification performance of the proposed model from a combined threshold-independent and class-specific perspective is presented in Table 4. As shown in Table 4a, the precision-recall curve has an average precision (AP) value of 0.8859, which suggests the model can keep a good balance between precision and recall for malignant instances. This result is particularly relevant for the present binary classification task, as the precision-recall analysis translates directly to the models ability to retrieve malignant samples, while controlling false-positive predictions.

**Table 4:** Classification performance of the proposed model on the test set (LIDC-IDRI dataset): **(a)** precision–recall curve, **(b)** receiver operating characteristic (ROC) curve, **(c)** confusion matrix and **(d)** calibration curve.

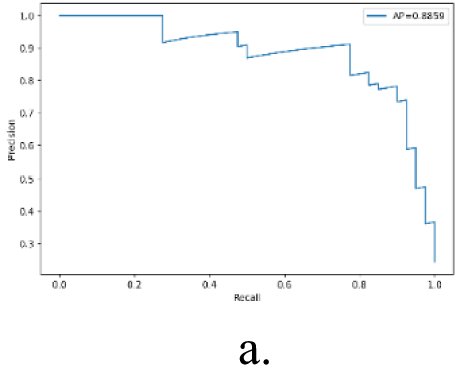	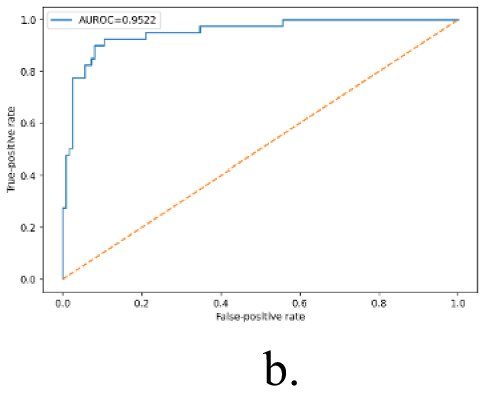	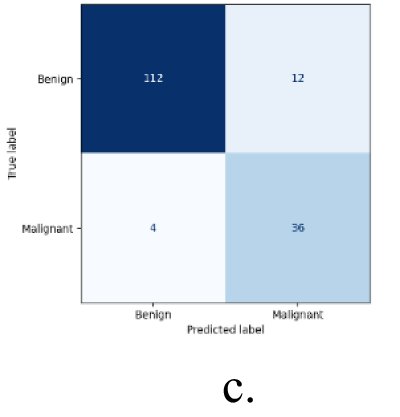	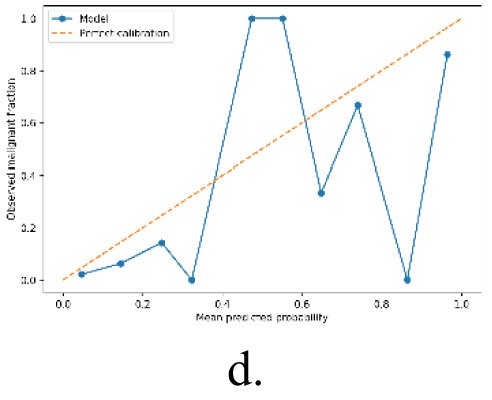

The ROC analysis in Table 4b further proves the high discriminative ability with AUROC of 0.9522. The ROC curve is significantly above the diagonal of random classification in the range of operation that we tested, which shows that the proposed model can effectively discriminate between benign and malignant cases at different decision thresholds. AUROC of more than 0.95 suggests that the learned representation is offering a high level of class separability rather than a specific classification threshold.

A confusion matrix providing a combined sample level evaluation of the final predictions is shown in Table 4c and calibration curve 4(d). 124 benign samples, 112 were correctly classified and 12 were wrongly predicted as malignant. Likewise, the method correctly identified 36 of 40 malignant samples, with only 4 malignant samples misidentified as benign. These predictions correspond to a total test accuracy of about 90.24%, a sensitivity of the malignant class of 90.00% and a specificity of the benign class of about 90.32%. The near equal sensitivity and specificity show that the proposed model maintains balanced recognition between the two classes instead of favoring either benign or malignant category.

The high AUROC, good average precision and balanced sensitivity-specificity profile together indicate that the proposed model can robustly separate benign from malignant samples. Importantly, the combined results from the ROC curve, precision-recall curve and confusion matrix demonstrate that performance is supported not only by threshold-independent ranking ability but also by reliable class-level predictions at the chosen operating threshold.

### 4.4 Parameter sensitivity analysis

Parameter sensitivity analysis was carried out to the effect of the lag and lead time constants, T1and T2, on the classification performance. The analysis was performed to investigate (Quinton et al., 2024) if the proposed BT–LL framework is robust to moderate variations in these parameters or its performance is sensitive to a particular parameter combination. The values of T1 and T2 were varied in the predefined range of 0.1–0.3, while keeping the rest of the model architecture and experimental settings unchanged. The performance was evaluated by accuracy, precision, sensitivity (recall), F1-score, AUROC and Matthews correlation coefficient (MCC). The results are shown in Table 5.

**Table 5:** Parameter sensitivity analysis for different $T_{1}$ and $T_{2}$ combinations.

Parameters	Accuracy	Precision	Recall	F1-score	AUROC	MCC
T1 = 0.1, T2 = 0.1	0.90	0.77	0.85	0.81	0.94	0.74
T1 = 0.1, T2 = 0.2	0.92	0.88	0.80	0.84	0.92	0.79
T1 = 0.1, T2 = 0.3	0.90	0.76	0.87	0.81	0.92	0.75
T1 = 0.2, T2 = 0.2	0.89	0.77	0.77	0.77	0.94	0.70
T1 = 0.2, T2 = 0.3	0.91	0.86	0.77	0.81	0.94	0.76
T1 = 0.3, T2 = 0.1	0.91	0.82	0.82	0.82	0.94	0.76
T1 = 0.3, T2 = 0.2	0.92	0.84	0.82	0.83	0.94	0.78
T1 = 0.3, T2 = 0.3	0.92	0.86	0.82	0.84	0.95	0.79

The classification performance was relatively stable across the different parameter combinations tested. The accuracy ranged from 0.89 to 0.92 and the AUROC ranged from 0.92 to 0.95. F1-score ranged between 0.77 and 0.84 and MCC ranged between 0.70 and 0.79. These relatively small variations in accuracy and AUROC indicate that the proposed framework is not very sensitive to moderate changes in the lag and lead parameters.

Among the configurations reported, T1 = 0.3, T2 = 0.3 achieved accuracy of 0.92, F1-score of 0.84, AUROC of 0.95 and MCC of 0.79, which is one of the strongest overall combinations. Also, the configuration of T1 = 0.1, T2 = 0.2 achieved an accuracy of 0.92, the highest precision of 0.88 and F1-score of 0.84. These results show that similar classification performance is obtained for different parameter settings, not only at a single isolated operating point.

Overall, the results of the sensitivity analysis indicate that the BT–LL framework is quite robust to variations in T1 and T2 as shown in Table 6. However, the trade-off between benign and malignant classification performance varies across parameter combinations, indicating that the parameters affect this trade-off and should be chosen based on the validation set rather than the held-out test set.

**Table 6:** Parameter sensitivity AUC, ROC-AUC curves for different $T_{1}$ and $T_{2}$ combinations.

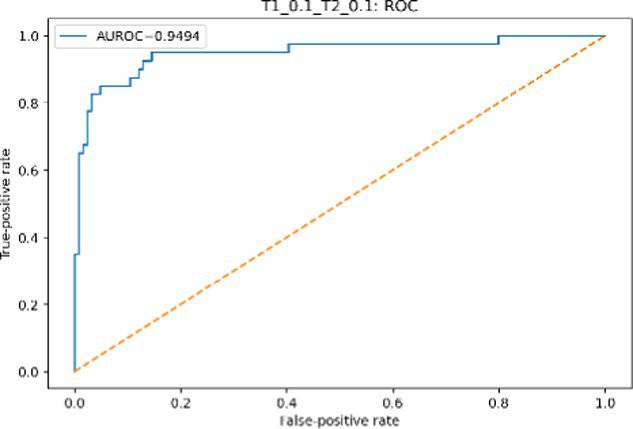	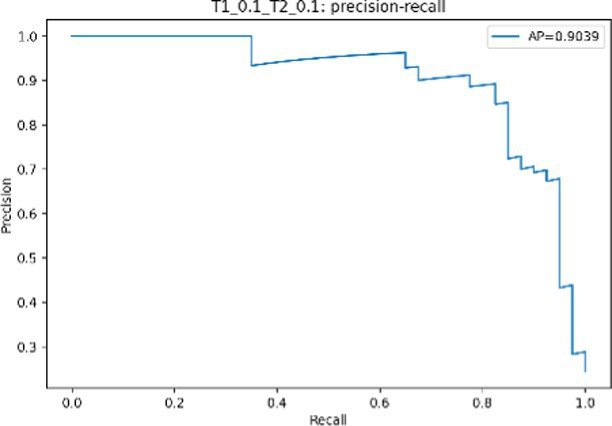	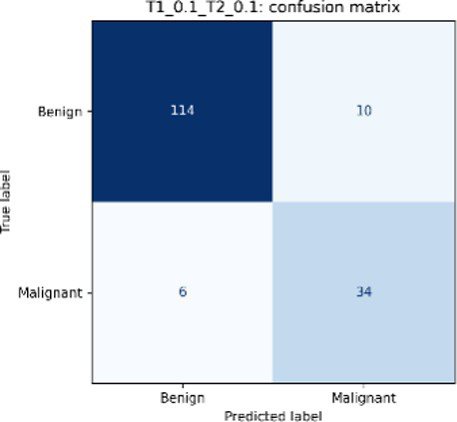
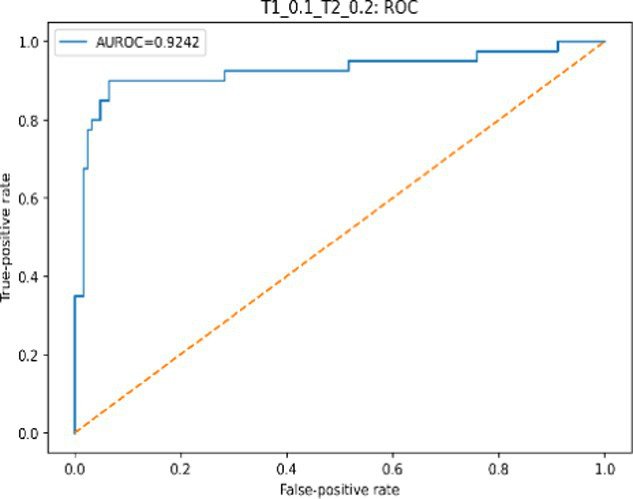	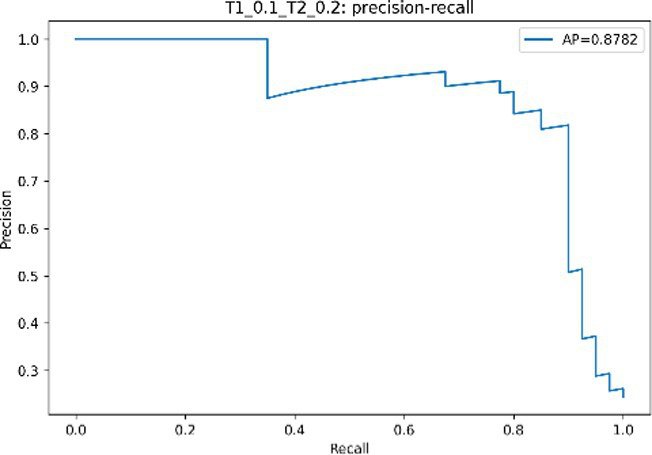	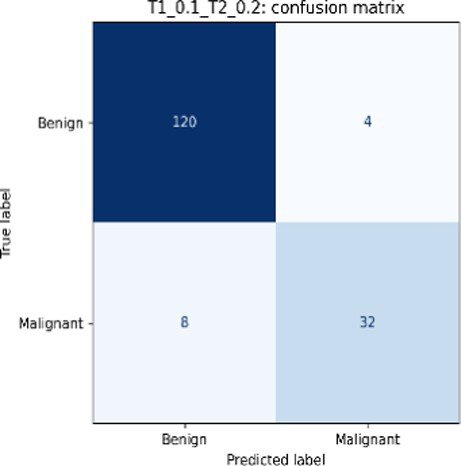
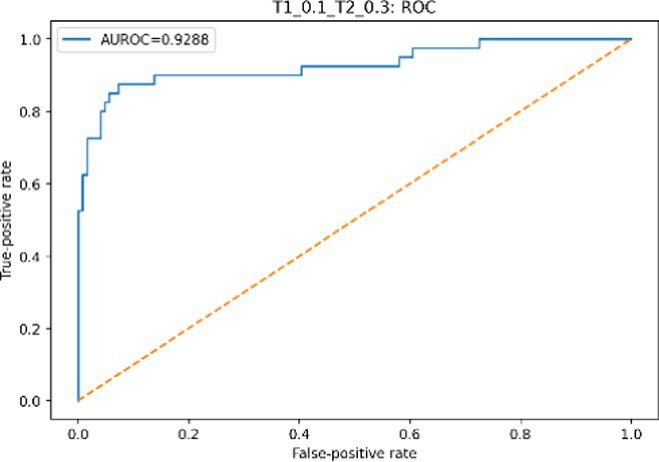	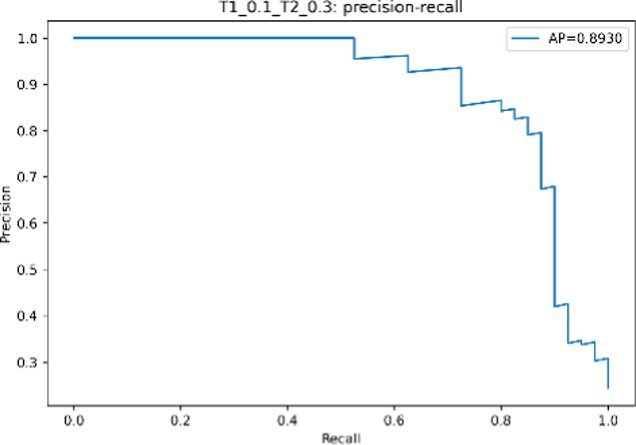	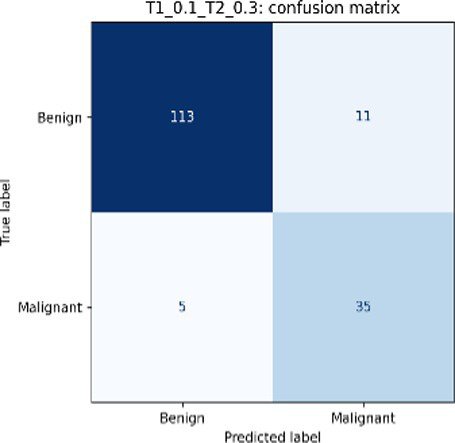
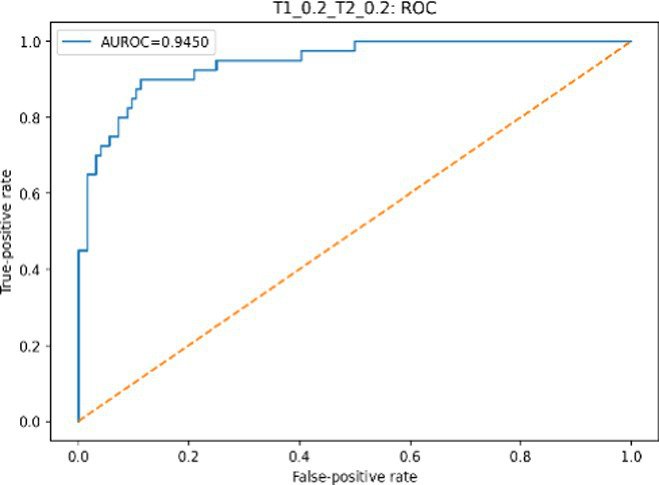	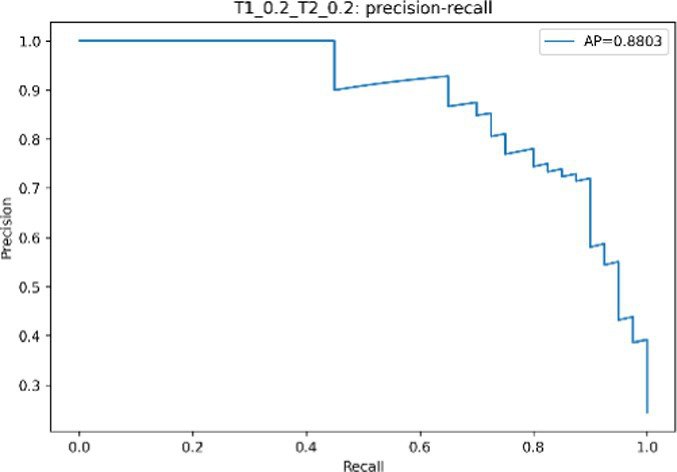	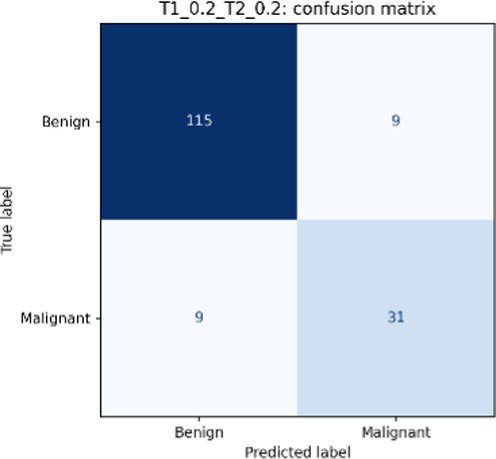
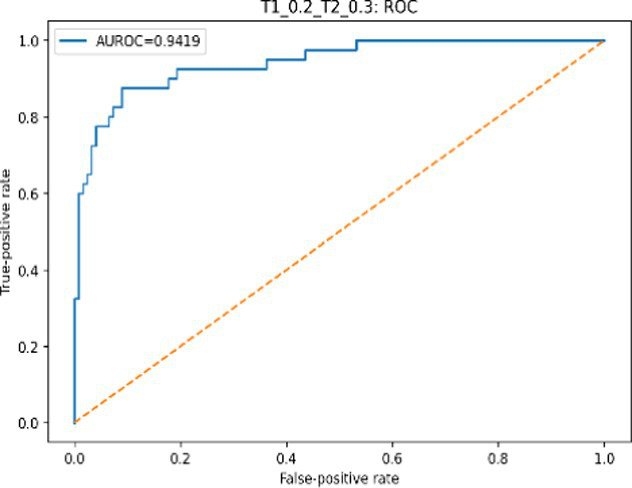	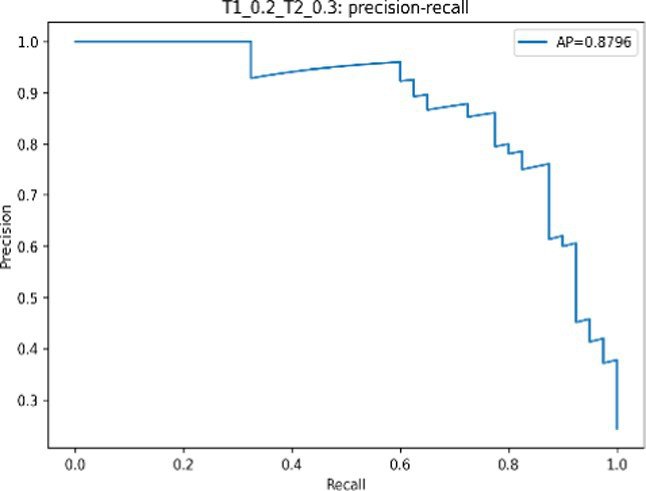	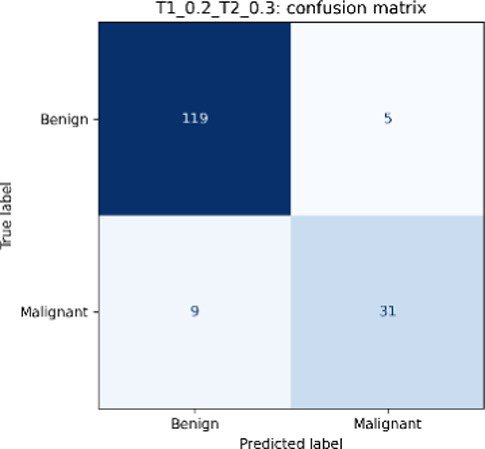
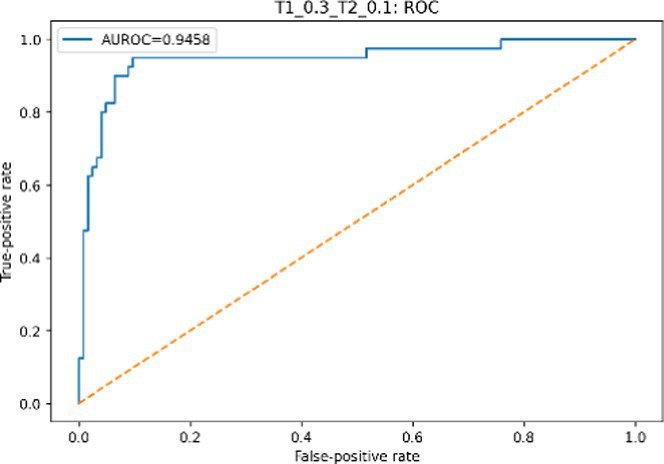	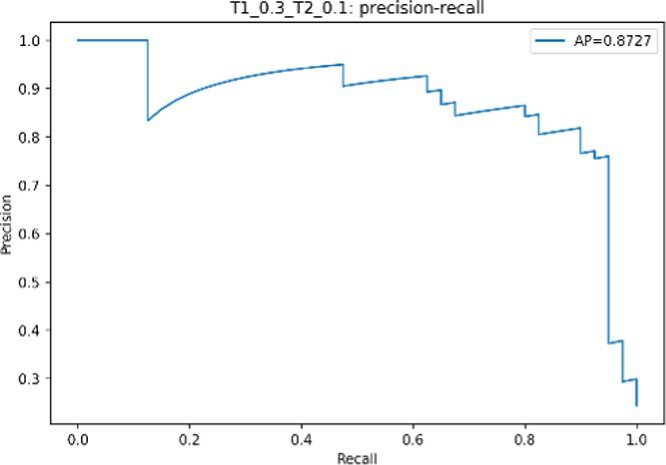	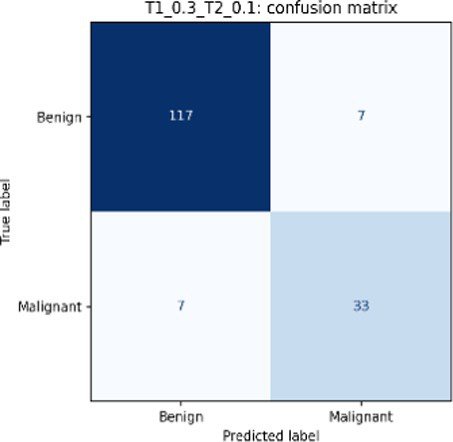
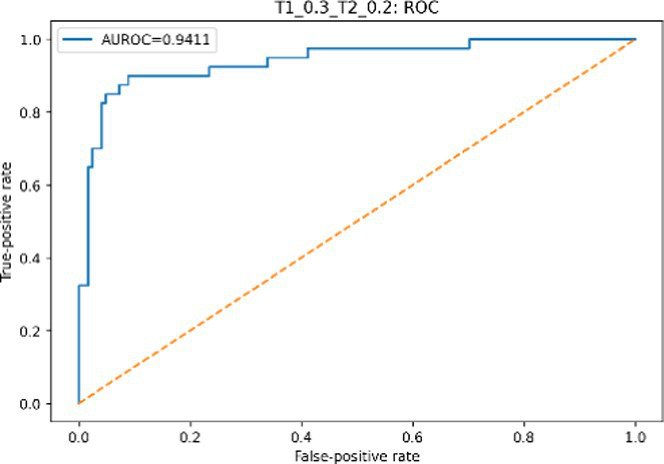	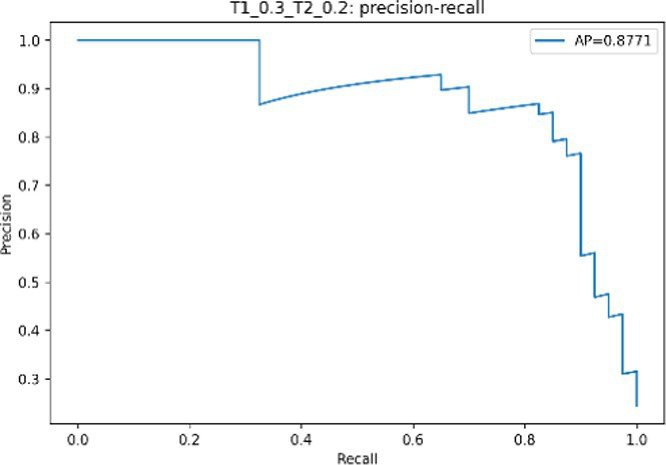	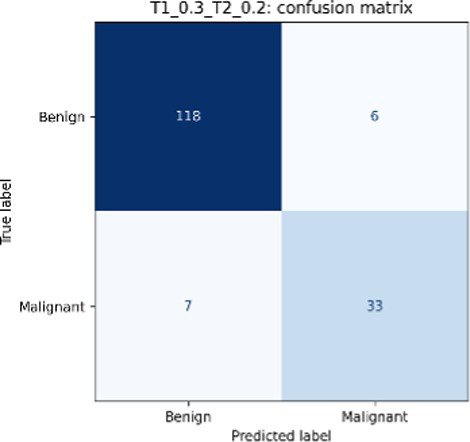
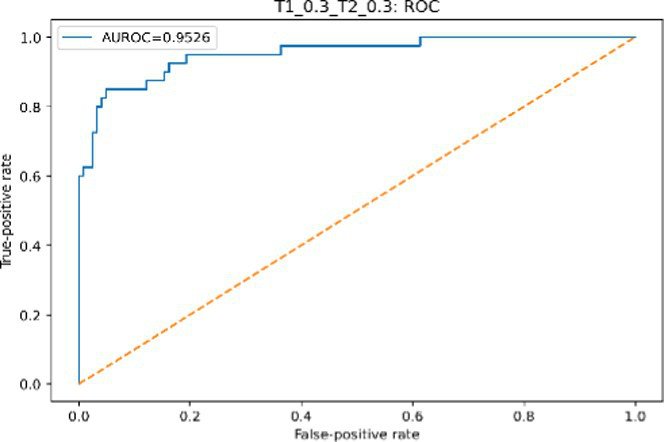	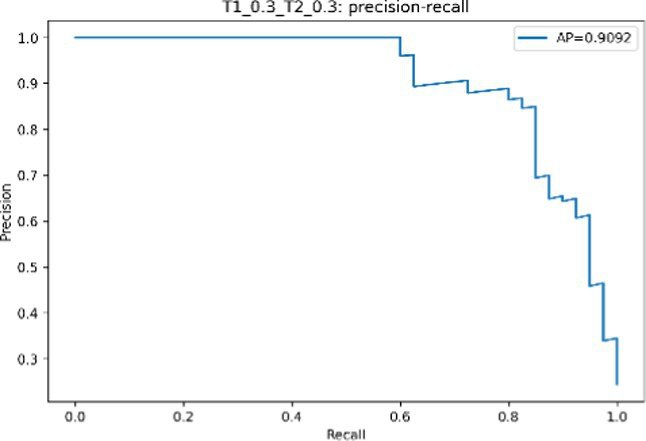	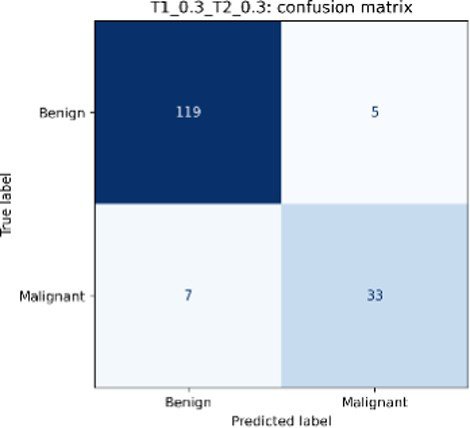

#### 4.4.1 Parameter robustness

The parameter-sensitivity analysis was conducted to determine whether classification performance depended strongly on a single choice of T1 and T2. In the revised patient-level experiments, the evaluated parameter combinations produced accuracies ranging from approximately 0.89 to 0.92, AUROC values from 0.92 to 0.95, F1-scores from 0.77 to 0.84, and MCC values from 0.70 to 0.79. The relatively narrow variation in accuracy and AUROC suggests that the proposed framework retains useful discrimination across moderate changes in the compensation parameters.

Nevertheless, the results also demonstrate that and are not functionally irrelevant. Differences in precision, sensitivity, F1-score, and MCC across the tested settings indicate that the compensation parameters influence the operating characteristics of the classifier. In particular, some configurations favored sensitivity, whereas others favored precision or overall discrimination. Thus, the parameter study supports relative robustness, but not complete parameter invariance.

The strongest reported parameter combinations achieved approximately 0.92 accuracy, with AUROC values reaching 0.95. Importantly, several distinct parameter settings produced similar performance, indicating that acceptable classification behavior was not confined to a single isolated operating point. This is desirable because a practically useful compensation framework should not depend on an extremely narrow choice of frequency-response parameters.

Accordingly, and should be selected using a predefined validation criterion rather than by maximizing performance on the held-out test set. The test set should remain reserved for final evaluation after parameter selection. The sensitivity results should therefore be interpreted primary report that the proposed frequency-compensation mechanism remains reasonably stable within the examined parameter range.

### 4.5 Cross validation analysis

Table 7 shows the Five-fold patient-level cross-validation was performed on the LIDC-IDRI dataset. Patients were assigned to folds such that no patient appeared simultaneously in the training, validation, and test subsets within a given fold. For each cross-validation iteration, the model was independently initialized and trained for 60 epochs, with one fold used for held-out evaluation and the remaining data used for model development. This procedure was repeated until each fold had served as the held-out test fold once. Predictions from all five held-out folds were then combined to obtain pooled out-of-fold performance estimates.

**Table 7:** Pooled five-fold out-of-fold performance with 95% bootstrap confidence intervals.

Metrics	Point_estimate	Lower_95	Upper_95
Accuracy	0.75	0.726013	0.781631
Balanced_accuracy	0.73	0.70035	0.764004
Precision	0.54	0.480968	0.59196
Sensitivity	0.68	0.628758	0.740995
Specificity	0.78	0.747368	0.812122
F1-score	0.60	0.554387	0.647982
MCC	0.43	0.373655	0.49482
AUROC	0.81	0.775394	0.838932
Average precision	0.67	0.612746	0.729193
Brier score	0.15	0.139882	0.165251

Ninety-five percent confidence intervals were estimated by bootstrap resampling at the patient level, thereby preserving within-patient dependence among multiple nodule images. Five-fold patient-level cross-validation was conducted to assess the robustness of the proposed framework. Each fold was trained independently for 60 epochs, with patient separation maintained between the model-development and held-out evaluation subsets within each iteration. Pooled out-of-fold predictions yielded an accuracy of 0.75 (95% CI, 0.726–0.782), balanced accuracy of 0.73 (0.700–0.764), sensitivity of 0.68 (0.629–0.741), specificity of 0.78 (0.747–0.812), F1-score of 0.60 (0.554–0.648), and MCC of 0.43 (0.374–0.495). Threshold-independent evaluation produced an AUROC of 0.81 (0.775–0.839) and average precision of 0.67 (0.613–0.729), while the Brier score was 0.15 (0.140–0.165). The total execution time for the five-fold cross-validation experiment was 4,924.07 s.

### 4.6 Explainability and qualitative analysis

The panels show the original CT slice image, class-specific Grad-CAM heatmap, Grad-CAM overlay, and input-gradient saliency map as shown in Figures 3a,b. Grad-CAM was generated from the top_conv layer of the proposed Bilinear Lag-Lead EfficientNetB3 model. The case was correctly classified as malignant with a predicted malignancy probability of 0.9659.

**Figure 3:**
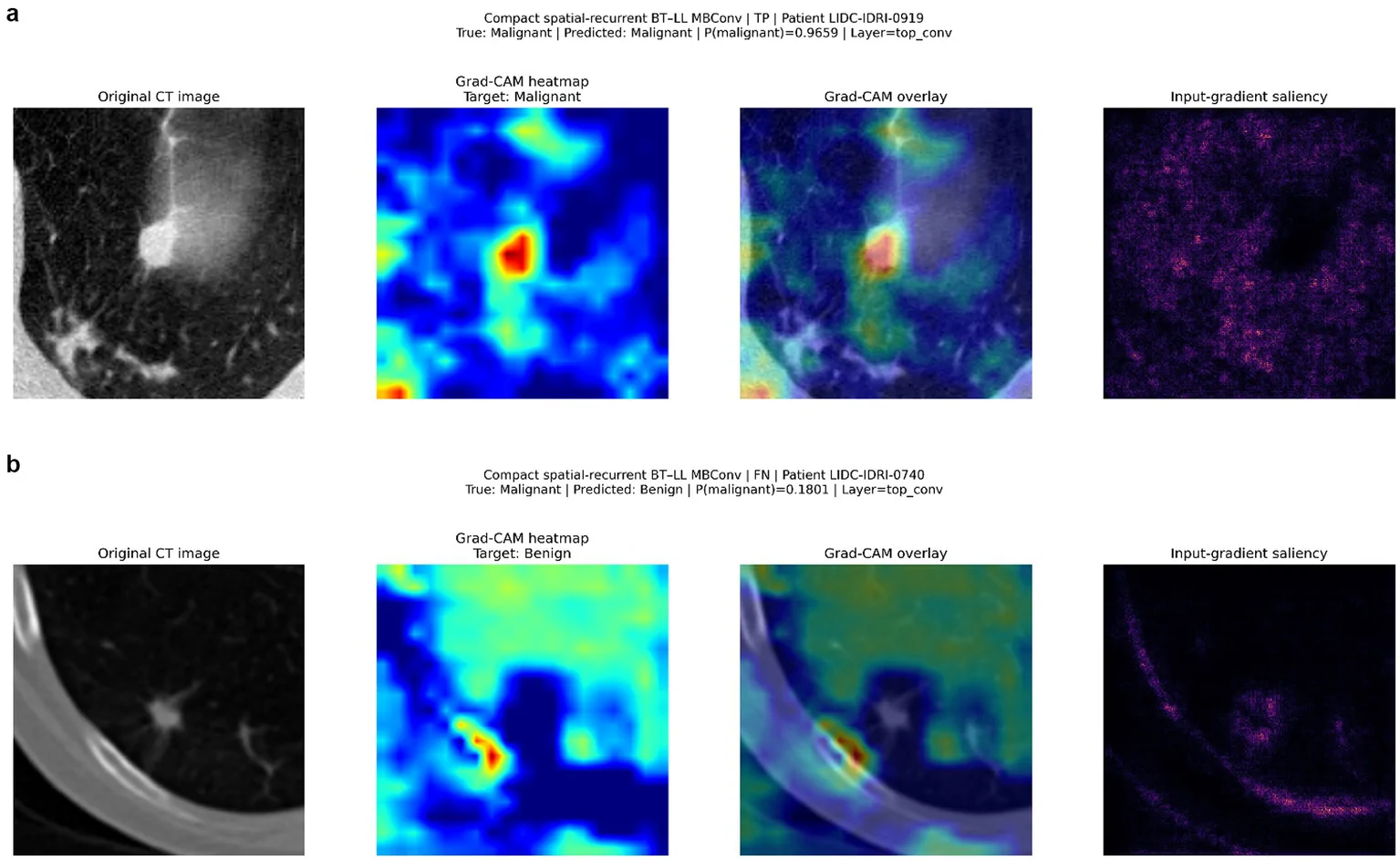
**(a,b)** Grad-CAM and saliency-based interpretation of a correctly classified malignant pulmonary nodule.

To evaluate model interpretability beyond intermediate feature-map inspection, Grad-CAM and input-gradient saliency analyzes were performed on representative test cases. Grad-CAM was generated from the top_conv feature layer and visualized together with the original CT image and model prediction. In correctly classified malignant cases, dominant activation was observed around the pulmonary nodule, while weaker responses were also present in surrounding anatomical structures. For LIDC-IDRI samples, classifier images were additionally matched to radiologist-annotated source slices and consensus nodule masks to examine correspondence between model activation and clinically defined lesion regions.

### 4.7 Explainability and radiological feature correspondence analysis

Nodule from LIDC-IDRI-0023, LIDC-IDRI-0267 and LIDC-IDRI-0420 the classifier input was registered to its corresponding source CT slice, and the radiologists consensus nodule mask was transferred to the classifier image as shown in Figures 4a–c. The high image-alignment score (0.984) confirms the reliable spatial correspondence between the input to the classifier and the source of the annotation. Radiologist spiculation rating was 5.0 for the nodule (median). The panels show classifier image, matched source slice with consensus nodule boundary and transferred mask overlayed on classifier image.

**Figure 4:**
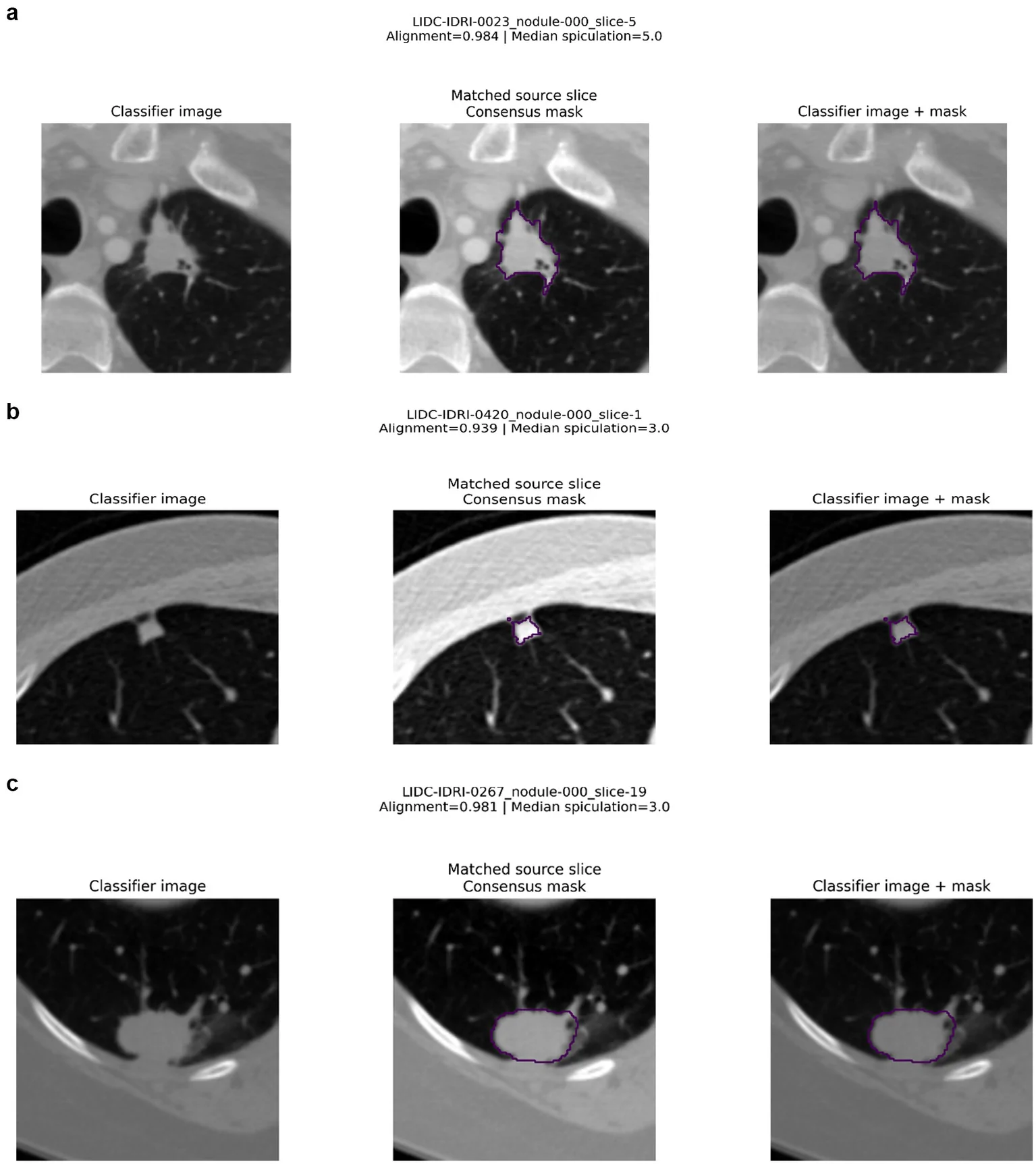
**(a–c)** Correspondence between classifier input and radiologist-annotated LIDC-IDRI lesion.

To integrate clinically relevant radiological information into the qualitative Grad-CAM evaluation (Alqhatni et al., 2025), the classifier inputs were linked to the corresponding source LIDC-IDRI slices and radiologist annotations. Consensus nodule masks were created from available radiologist segmentations and spatially registered to the classification images. A typical case where the classifier image was highly similar to the original annotated CT slice (alignment score = 0.984) is shown in Figure 4a. The corresponding nodule had a median spiculation score of 5.0, a feature of strongly expressed radiological margin. The consensus contour that was transferred to the classifier image accurately delineated the region of the lesion and provided a spatial reference for later comparison with model activation maps.

### 4.8 Frequency-response validation of the deployed bilinear Lag-Lead kernels

The preceding transfer-function analysis characterizes the intended continuous and bilinear-transform-derived Lag-Lead responses. However, the actual network implementation operates using compact 3 × 3 spatial FIR kernels. Therefore, the frequency responses of the deployed kernels were explicitly evaluated to establish the relationship between the theoretical compensator and its spatial realization.

Figure 5 compares the theoretical lag prototype, the exact digital response obtained after bilinear transformation, and directional responses of the deployed 3 × 3 FIR kernel. The continuous prototype and exact digital response closely coincide under the selected frequency mapping, whereas the compact deployed FIR exhibits a shallower attenuation profile. Along the spatial-frequency axis, the deployed response decreases gradually toward higher frequencies, while the diagonal response exhibits stronger attenuation. Thus, the deployed kernel preserves the intended low-frequency-preserving tendency of the lag prototype but does not reproduce its full high-frequency attenuation exactly.

**Figure 5:**
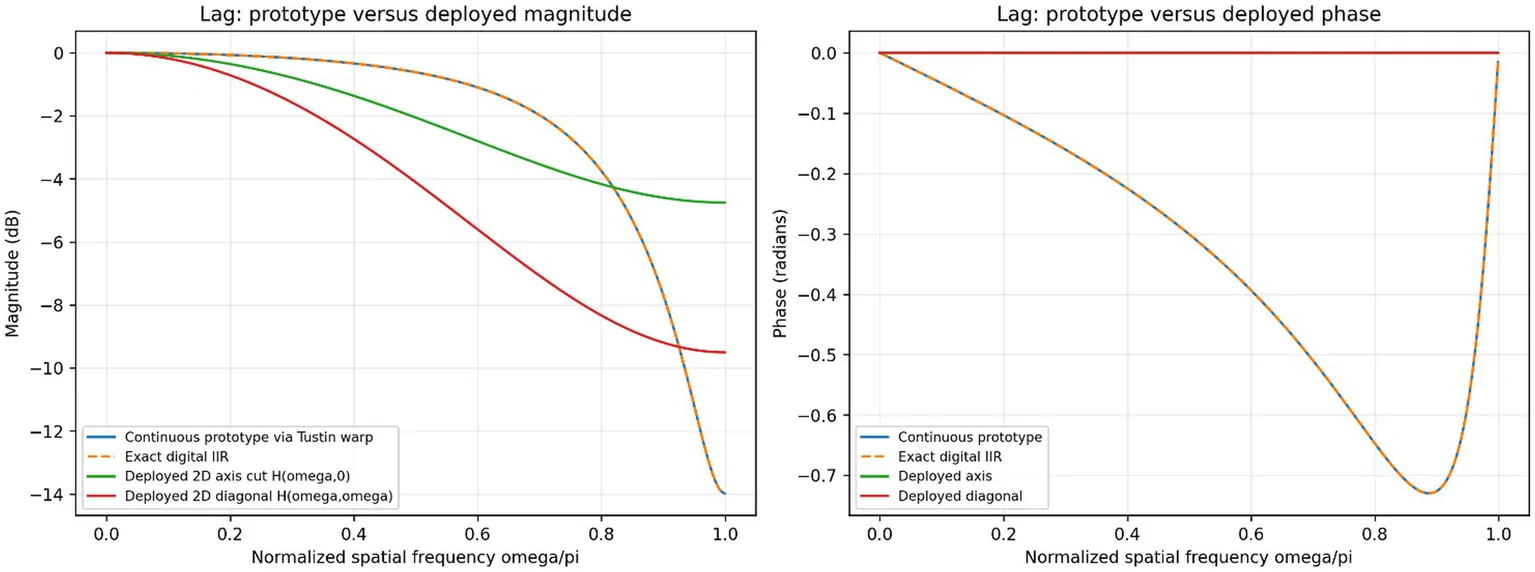
Comparison of the theoretical lag prototype and deployed 3 × 3 response.

The theoretical/digital lag response exhibits a non-zero frequency-dependent phase characteristic, whereas the deployed symmetric FIR kernel exhibits approximately zero phase. This difference is expected from the centered symmetric spatial FIR construction and confirms that the deployed kernel should be interpreted as a compact magnitude-response approximation rather than an exact dynamical realization of the original first-order compensator. The full two-dimensional DTFT further demonstrates that the deployed lag kernel has maximum gain around zero spatial frequency and progressively lower magnitude toward higher spatial frequencies. The attenuation also exhibits directional variation between the coordinate axes and diagonal directions, which is not visible in the original one-dimensional prototype analysis as shown in Figure 6.

**Figure 6:**
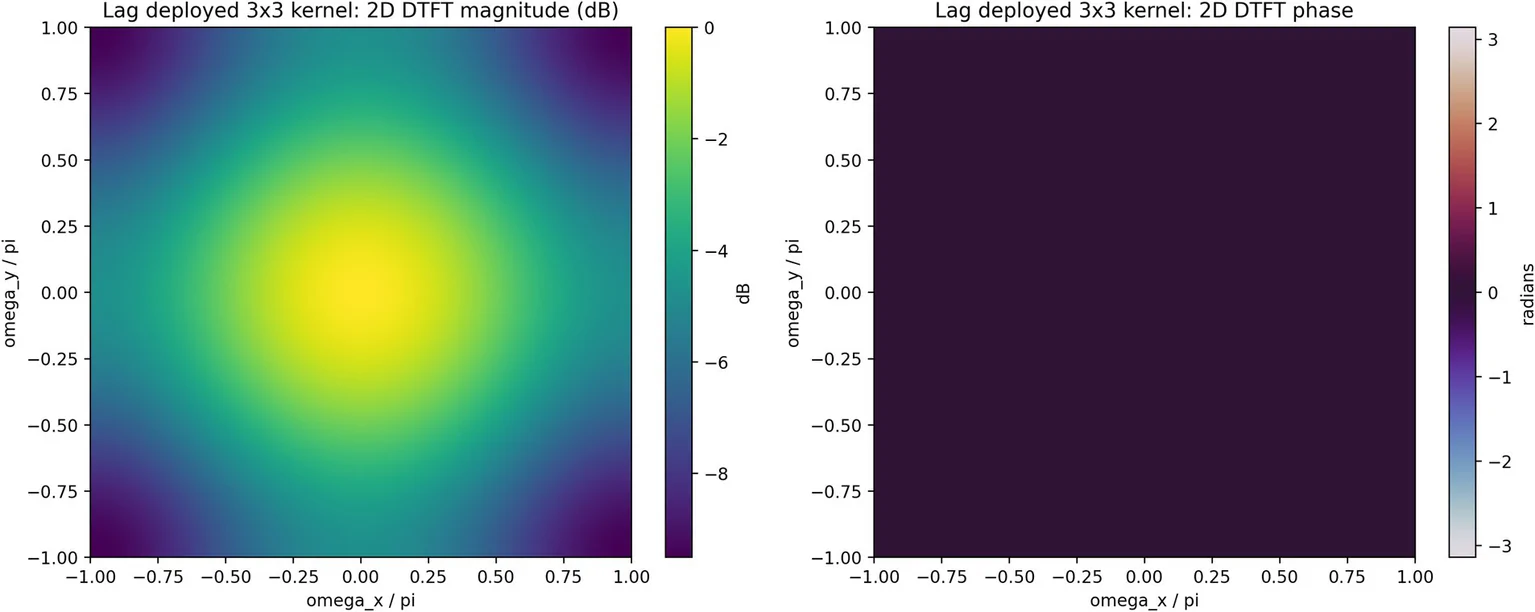
Two-dimensional DTFT magnitude and phase responses of the deployed 3 × 3 lag FIR kernel.

For the lead branch, the deployed FIR retains the intended tendency toward increasing gain with spatial frequency as explained in Figure 7. Nevertheless, the compact FIR response deviates from the exact digital prototype at higher frequencies. In particular, the theoretical response continues to increase toward the Nyquist limit, whereas the deployed approximation exhibits a lower saturated gain. Therefore, the lead FIR is likewise treated as a compact spatial approximation preserving the principal high-frequency-enhancement tendency rather than an exact implementation of the prototype.

**Figure 7:**
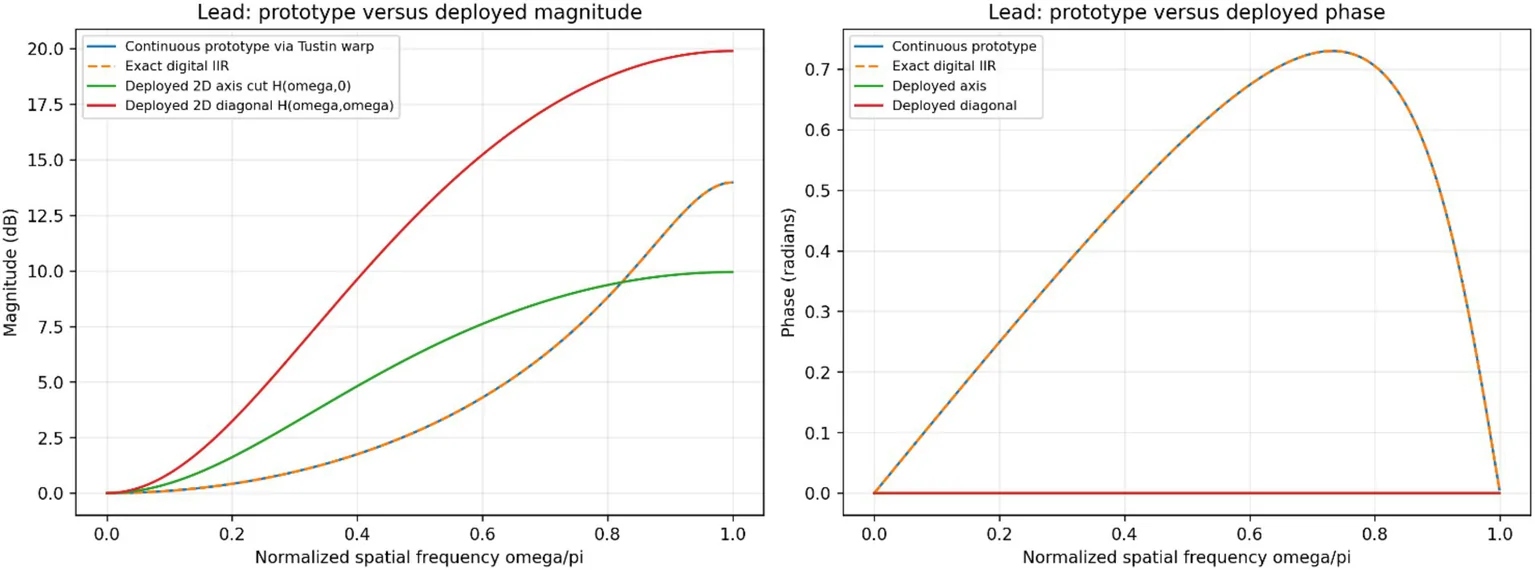
Comparison of the theoretical led prototype and deployed 3 x 3 response.

The prototype-to-implementation mapping is now explicitly verified at three levels. First, the continuous lag/lead prototypes are transformed to exact digital responses using the bilinear transformation. Second, compact symmetric FIR kernels are obtained for spatial deployment. Third, the actual two-dimensional DTFT magnitude and phase responses of those deployed kernels are computed directly from their numerical 3 × 3 coefficients. Directional axis and diagonal cuts of the 2D responses are further plotted alongside the prototype/digital responses to show both their correspondence and their approximation error as shown in Figure 8.

**Figure 8:**
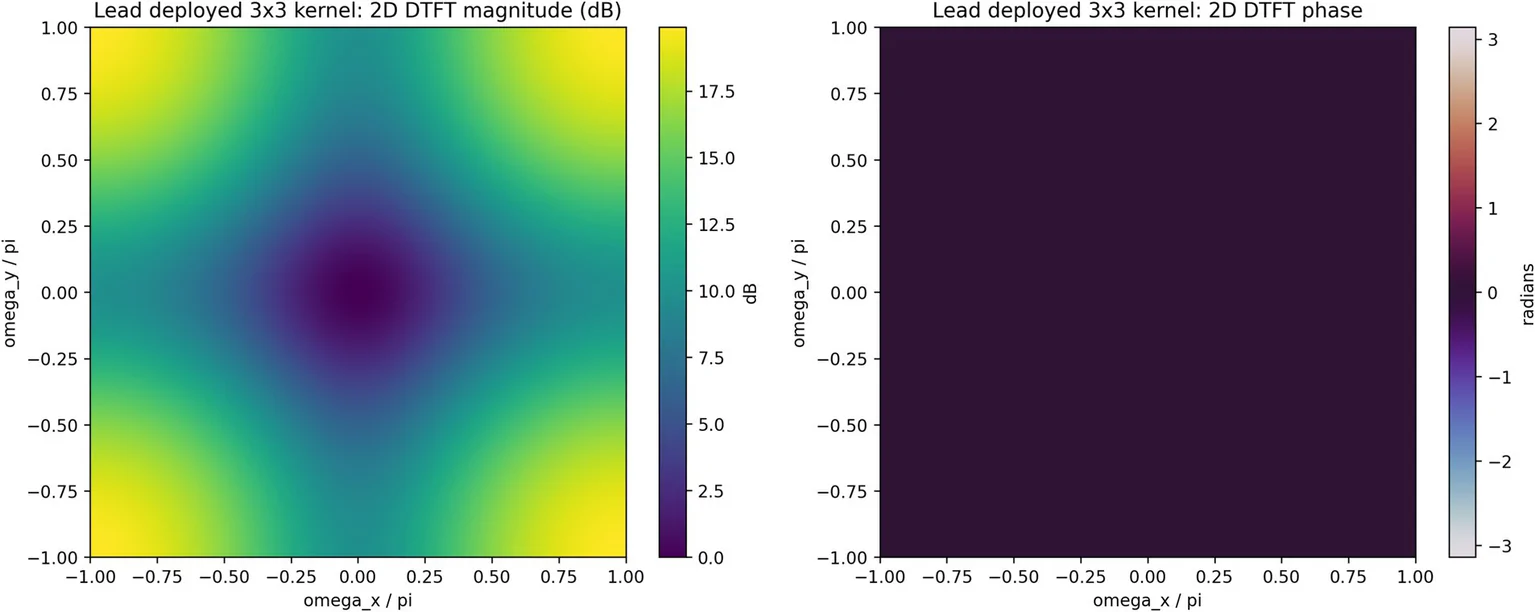
Two-dimensional DTFT magnitude and phase responses of the deployed 3 × 3 lead FIR kernel.

The revised results demonstrate that the deployed kernels retain the intended qualitative frequency-selective behavior but do not exactly reproduce the entire prototype response. Specifically, the deployed lag kernel retains stronger response to lower spatial frequencies, whereas the lead compensation provides relatively greater emphasis to higher spatial frequencies. The symmetric FIR realization has approximately zero phase, which differs from the phase behavior of the original digital IIR prototype. These implementation-level results are now explicitly discussed, and the terminology throughout the manuscript has been revised to avoid implying exact equivalence between the continuous compensators and the deployed 3 × 3 spatial kernels.

## 5 Discussion

In this work, a frequency response BT-LL feature-compensation framework with EfficientNetB3 was proposed for benign-malignant pulmonary nodule classification. As opposed to the exploratory experiments in the original evaluation, the new evaluation was performed on patient level data and used an untouched held-out test set, decreasing the risk of correlated nodules or images from the same patient contributing to both model development and final evaluation.

Results indicate that Lag and Lead compensations have different impacts on classification behavior. The Lag branch was designed to preserve slowly varying structural information, while the Lead branch was designed to preserve boundary and detail related information. Together, the two produced a sensitivity-oriented operating point with balanced discrimination of benign and malignant samples but did not outperform each individual ablation on all metrics. This distinction matters because the statistical analyzes did not reveal a significant superiority of the full model after correcting for multiple comparisons. Therefore, the contribution of the proposed framework should be better understood in terms of controlled frequency feature refinement, combined operating characteristics, and explicit implementation-level interpretability, rather than consistent superiority across all metrics.

### 5.1 Comparison of the proposed model with existing deep-learning methods

Table 8 places the proposed framework in the context of previously reported deep-learning approaches for pulmonary nodule classification. Direct numerical ranking of these methods should be interpreted cautiously because the reported studies may differ in dataset composition, sample size, preprocessing, labeling criteria, and train-validation-test partitioning. Accordingly, the table is intended to provide literature context rather than a head-to-head performance comparison.

**Table 8:** Performance comparison of the proposed model with existing deep-learning methods.

Methods	Precision	Recall	F1-score	Accuracy	Evalution protocol
CNN (Salhi et al., 2026)	0.99	0.97	0.98	0.98	High/SliceLevel
DCAI (Wang S. et al., 2025)	0.95	0.97	0.96	0.96	Slice Level
SSTL-FM	0.94	0.92	0.93	0.93	High/SliceLevel
Proposed model	**0.89**	**0.90**	**0.81**	**0.90**	Patient-level

Bold values represent the metric values obtained by the proposed model using patient-level splitting.

Several previously reported approaches achieved high classification scores under their respective experimental protocols. However, when image- or slice-level partitioning is used, multiple observations originating from the same patient may potentially occur in different data subsets unless explicit patient grouping is enforced. Such a protocol can produce optimistic performance estimates because anatomically correlated samples from the same subject may be represented in both training and evaluation data. For this reason, the present study adopted patient-level partitioning, ensuring that all samples from an individual patient were assigned exclusively to the training, validation, or test subset.

Under this stricter patient-separated evaluation, the proposed model achieved an accuracy of 0.90, sensitivity of 0.90, precision of 0.89, and F1-score of 0.81 on the held-out test set. Although some literature-reported methods show higher numerical accuracy, these values cannot be interpreted as direct results of superiority because the underlying evaluation protocols differ. The principal methodological advantage of the present evaluation is therefore not a higher isolated accuracy value, but the use of patient-level separation, validation-based model selection, and an untouched held-out test set to provide a more conservative assessment of generalization.

### 5.2 Computational complexity and efficiency analysis

Table 9 summarizes the computational complexity of the EfficientNetB3 baseline and the proposed BT-LL stage configurations [(Stage1 S1), (Stage2 S2), (Stage3 S3), and (Stage4 S4)] in terms of the number of parameters and GFLOPs. All configurations were evaluated using the same input resolution, so the values provide a direct comparison of the additional computational burden introduced by the compensation modules.

**Table 9:** Computational complexity of the EfficientNetB3 baseline and stage-wise BT–LL configurations.

Configuration	Active stages	Parameters (M)	GFLOPs
EfficientNetB3	None	11.24	4.004
S1	S1	11.24	4.046
S2	S2	11.24	4.027
S3	S3	11.29	4.046
S4	S4	18.34	5.426
S1 + S2	S1 + S2	11.25	4.069
S2 + S3	S2 + S3	11.30	4.069
S1 + S2 + S3	S1 + S2 + S3	11.31	4.111
Proposed	**S1 + S2 + S3 + S4**	**18.41**	**5.533**

Bold values represent the parameters and GFLOPs values obtained by the proposed model’s combined stages.

EfficientNetB3 baseline model required 11.24 M parameters and 4.004 GFLOPs. The individual introduction of BT-LL compensation at early and intermediate stages caused a small increase in complexity. The S1 and S2 configurations had around 11.24 million parameters and 4.046 and 4.027 GFLOPs, respectively. The S3 configuration had 11.29 million parameters and 4.046 GFLOPs. Likewise, the multi-stage configurations, S1 + S2, S2 + S3 and S1 + S2 + S3, remained computationally efficient with 4.069–4.111 GFLOPs and approximately 11.25–11.31 million parameters.

A larger increase was observed when compensation was introduced at S4. The S4-only configuration required 18.34 million parameters and 5.426 GFLOPs. This increase is primarily associated with applying the compensation and learned fusion operations to the high-dimensional feature representation at the final EfficientNetB3 stage. Consequently, configurations involving S4 incur a greater parameter and computational cost than those restricted to earlier stages.

The complete proposed configuration, in which BT-LL compensation is applied at S1 + S2 + S3 + S4, required 18.41 million parameters and 5.533 GFLOPs. Although this represents an increase over the EfficientNetB3 baseline, the computational requirement remains moderate for a multi-stage feature-compensation architecture. The additional complexity results from the fixed spatial filtering, lag/lead compensations, and learned feature-fusion operations introduced at the selected stages.

Overall, the analysis demonstrates that early- and intermediate-stage BT-LL modules introduce relatively little computational overhead, whereas inclusion of the S4 module accounts for most of the additional model complexity. This provides an explicit trade-off between computational cost and the broader multi-stage representation used by the complete proposed architecture.

### 5.3 Ablation study analysis

A comprehensive ablation analysis was conducted to investigate the contribution of the proposed Lag-Lead compensation mechanism (Li and Janson, 2024; Meyes et al., 2019) and to determine the design choices responsible for the final classification performance. Specifically, three combined experiments were performed: (i) evaluating the effect of inserting the Lag-Lead compensator at different feature-extraction stages, (ii) examining the robustness of the model under different class-imbalance ratios, and (iii) independently evaluating the contributions of the Lag and Lead components against their combined formulation. These experiments isolate the influence of architectural placement, data imbalance, and compensation strategy on the overall performance of the proposed framework.

#### 5.3.1 Effect of Lag-Lead compensator insertion stage

The first ablation experiment investigates the influence of the insertion position of the Lag-Lead compensator within the feature-extraction network. The compensator was introduced at different stages, denoted as (Stage1 S1), (Stage2 S2), (Stage3 S3), and (Stage4 S4), and their combinations were systematically evaluated while maintaining the remaining training and evaluation settings unchanged. This experiment was designed to determine whether frequency compensation is more beneficial at a particular representation level or when progressively incorporated across multiple stages. The results in Table 10 demonstrate that the effectiveness of BT-LL compensation depends on both its insertion location and the number of compensated feature stages. The S1 configuration achieved the highest performance on several individual metrics, including an accuracy of 0.94, specificity of 0.95, F1-score of 0.88, and MCC of 0.85, while maintaining a sensitivity of 0.90 and AUROC of 0.96. Thus, S1 represents the most favorable accuracy-complexity trade-off among the evaluated configurations on the present held-out test partition.

**Table 10:** Ablation study: test performance of stage configurations.

Configuration	Test Acc.	Sensitivity	Specificity	F1-score	MCC	AUROC	Parameters	Training time (s)
EfficientNetB3	0.90	0.87	0.91	0.81	0.75	0.93	11,236,400	787.96
S1	0.94	0.90	0.95	0.88	0.85	0.96	11,240,080	1337.66
S1 + S2 + S3	0.89	0.82	0.91	0.78	0.71	0.95	11,305,976	534.15
S2	0.91	0.85	0.93	0.82	0.77	0.95	11,244,224	727.74
S3	0.91	0.75	0.96	0.81	0.76	0.93	11,294,472	701.98
S2 + S3	0.94	0.85	0.97	0.88	0.84	0.96	11,302,296	595.40
S4	0.92	0.77	0.97	0.83	0.79	0.95	18,343,472	935.30
S1 + S2	0.91	0.82	0.94	0.82	0.76	0.95	11,247,904	598.24
Proposed work	**0.90**	**0.90**	**0.90**	**0.81**	**0.80**	**0.96**	**18,413,048**	**608.30**

Bold values represent the metric values obtained by the proposed model.

The full S1 + S2 + S3 + S4 configuration was retained as the complete proposed architecture rather than single-metric reasons. The objective of the proposed BT-LL framework is to perform frequency-response refinement throughout the hierarchical feature-extraction process. Compensation at S1 alone evaluates the effect of frequency refinement at an early representation level, whereas the full configuration implements the proposed mechanism across all four feature stages, allowing low-level structural, intermediate textural, and deeper discriminative representations to be refined within a single unified architecture. Because the fusion operation is learned independently at each compensated stage, each stage can adapt the relative contribution of the original, low-frequency, and edge-sensitive representations according to the characteristics of that level.

#### 5.3.2 Effect of class-imbalance ratio

The second experiment evaluates the robustness of the proposed model under different class-imbalance conditions. Different benign-to-malignant sample ratios were considered to investigate whether the observed performance depends strongly on a particular class distribution. This analysis is particularly important because conventional accuracy can provide an overly optimistic assessment when one class dominates the dataset.

As reported in Tables 11, 12, the proposed model maintains competitive performance across the investigated imbalance ratios. Although variations in class distribution produce corresponding changes in individual metrics, the model preserves a favorable balance between malignant-class sensitivity and benign-class specificity. The AUROC, average precision, balanced accuracy, F1-score, and MCC further demonstrate that the classification capability is not solely driven by the majority class.

**Table 11:** Performance of the proposed model under different benign-to-malignant training ratios.

Training ratio (benign: malignant)	Accuracy	Sensitivity	F1-score	Precision	MCC	AUROC
Natural (2.64:1)	**0.90**	**0.90**	**0.81**	**0.92**	**0.80**	**0.96**
1:1	0.90	0.87	0.82	0.90	0.76	0.94
2:1	0.89	0.85	0.80	0.90	0.73	0.94
3:1	0.90	0.72	0.79	0.90	0.74	0.95

Bold values represent the metric values obtained with the training ratio used in the proposed model.

**Table 12:** Sensitivity analysis of the proposed model under different class-imbalance ratios.

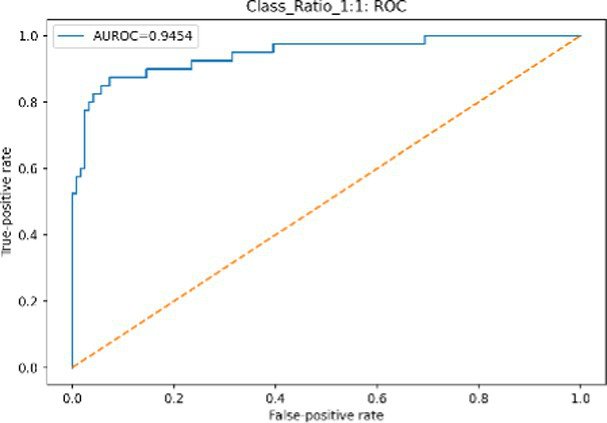	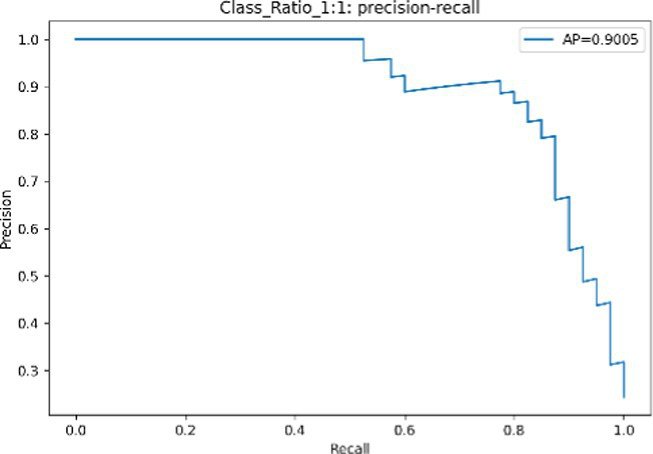	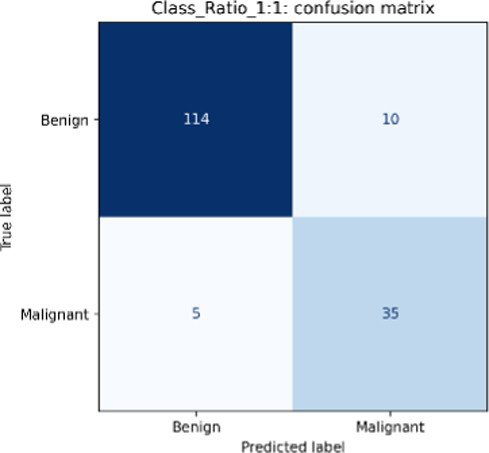
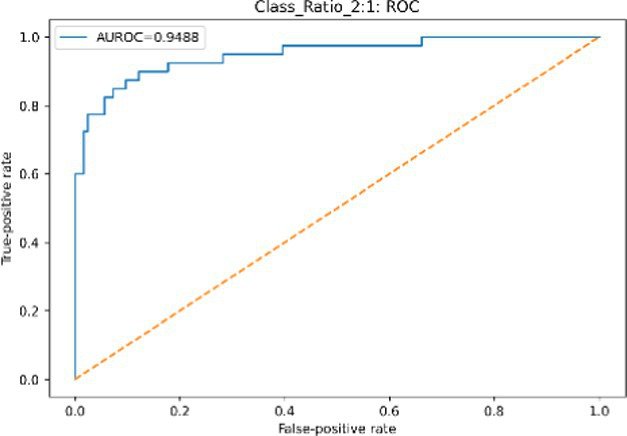	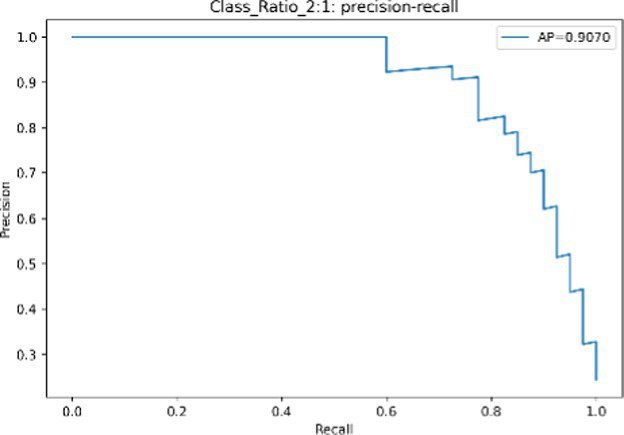	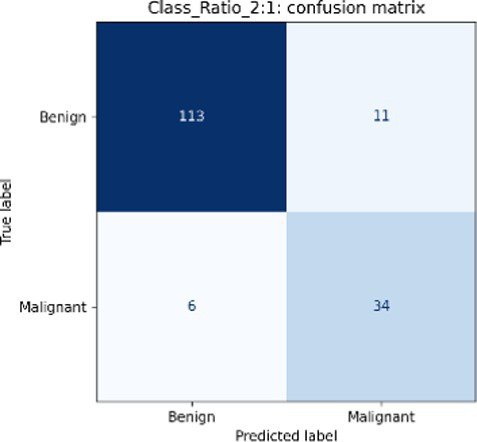
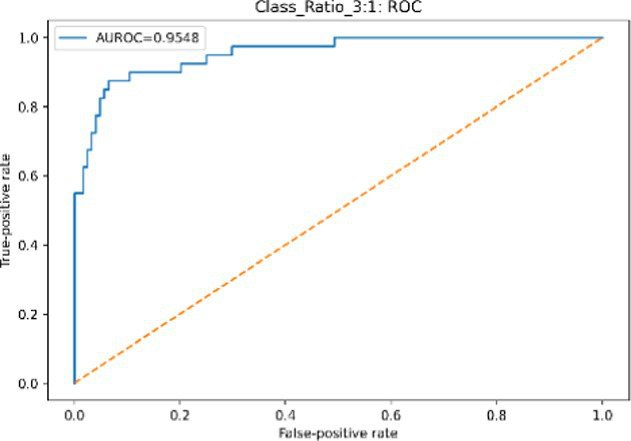	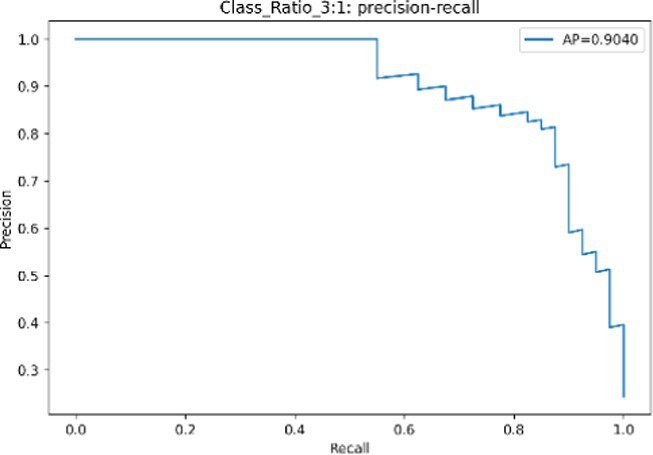	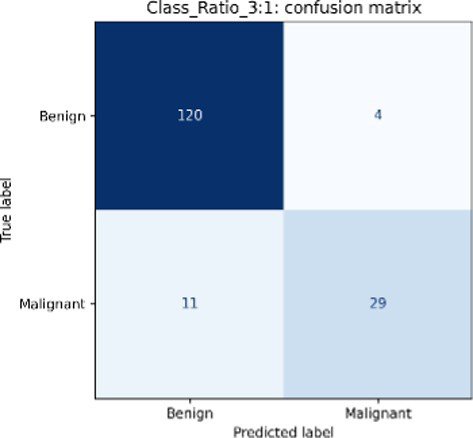

More importantly, the comparatively stable performance across different ratios demonstrates that the proposed feature-compensation strategy remains effective under varying class distributions. This observation provides additional results that the improvements achieved by the proposed framework are attributable to its learned discriminative representations rather than merely to the composition of the training samples.

#### 5.3.3 Impact of lag and lead compensation

The third ablation experiment shows the contributions of Lag and Lead compensation branches both independently and jointly in Table 13. The Lag-only, Lead-only, proposed combined Lag-Lead and the proposed model without spatial filtering were assessed using the same experimental protocol. Additionally, the original EfficientNetB3 model was included for reference. The aim of this experiment was to investigate the impact of the individual frequency-compensation branches on the classification performance and if their ensemble can achieve a better discrimination between benign and malignant nodules.

**Table 13:** Ablation analysis of lag-only, lead-only, and proposed Lag-Lead compensation.

Model name	Precision	Recall	F1-score	Accuracy	Confusion matrix
EfficientNetB3	0.88	0.87	0.81	0.90	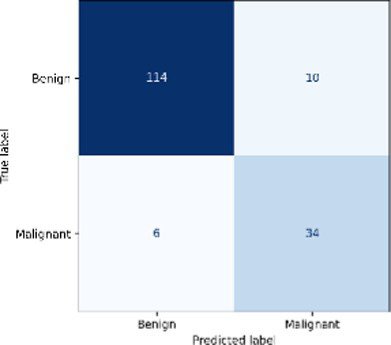
Lag compensation	0.82	0.85	0.84	0.92	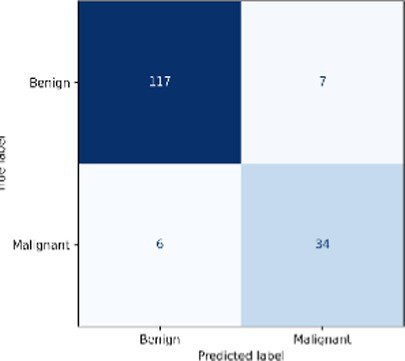
Lead compensation	0.91	0.77	0.83	0.92	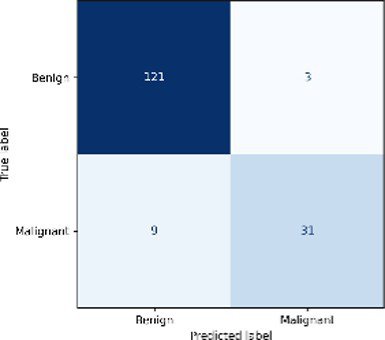
Proposed model without spatial filters	0.90	0.67	0.77	0.90	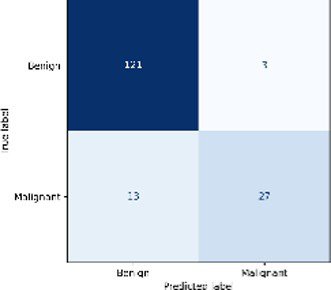
Proposed model	**0.89**	**0.90**	**0.81**	**0.90**	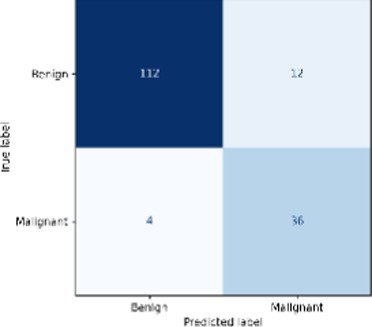

Bold values represent the metric values obtained by the proposed model by combining all the components.

The Lag branch is designed to preserve relatively smooth and slowly varying feature information, thus retaining broader structural and contextual characteristics. Conversely, the Lead branch is designed to respond to rapidly changing features that correspond to boundaries, local transitions, and fine structural details. Thus, the two branches are designed to operate on distinct aspects of the extracted feature representation.

The Lag-only model achieved an accuracy of 92.%, a sensitivity of 85%, a specificity of 94% and an F1-score of 84% (Table 13). The Lead-only configuration showed a slightly higher accuracy of 92% and specificity of 97%, but the sensitivity was lowered to 77%. The combined Lag-Lead model however attained the highest sensitivity of 90% and balanced accuracy of 90%, which indicates a more balanced ability to identify malignant and benign cases. It achieved an overall accuracy of 90%, an F1-score of 83% and an AUROC of 95%.

Eliminating the spatial-filtering components yielded an accuracy of 90% and a high specificity of 97% but a significant drop in sensitivity to 67%. This observation indicates that the spatial compensation branches are useful for the malignant-case detection at the selected operating point. The model without spatial filters had a better AUROC, but its considerably lower sensitivity indicates that threshold-independent discrimination is not a complete representation of the clinically relevant operating-point performance.

Overall, the ablation results show that Lag and Lead branches have different performance characteristics. The Lag-only configuration yields relatively higher sensitivity, while the Lead-only configuration favors specificity. Their combination gives the highest sensitivity and balanced accuracy among the branch-based configurations, indicating that the two components have combined behavior rather than identical contributions. However, since the paired statistical comparisons did not reveal statistically significant differences, these results should be interpreted as trends in performance rather than statistically confirmed superiority.

#### 5.3.4 Statistical significance analysis of ablation results

To determine whether the numerical differences observed among the ablation configurations were statistically supported, paired statistical analyzes were performed because all models were evaluated on the same held-out test cases. Cochran’s Q test was first used as an omnibus test of classification correctness across the compared configurations. Pairwise differences between the Full Proposed model and each component-ablation variant were subsequently assessed using the exact McNemar test, with Holm correction applied for multiple comparisons as shown in Table 14.

**Table 14:** Patient-level clustered bootstrap comparison of component-ablation configurations (5,000 replicates).

Comparison	Metric	Proposed	Comparison	Difference (full − other)	95% clustered-bootstrap CI
Proposed vs. Lag only	Balanced Accuracy	0.8802	0.8968	−0.0165	−0.0625 to 0.0305
	Sensitivity	0.8250	0.8500	−0.0250	−0.1081 to 0.0588
	Specificity	0.9355	0.9435	−0.0081	−0.0513 to 0.0317
	AUROC	0.9522	0.9423	+0.0099	−0.0130 to 0.0383
Proposed vs. Lead only	Balanced Accuracy	0.8802	0.8754	+0.0048	−0.0308 to 0.0452
	Sensitivity	0.8250	0.7750	+0.0500	0.0000 to 0.1250
	Specificity	0.9355	0.9758	−0.0403	−0.0840 to −0.0079
	AUROC	0.9522	0.9361	+0.0161	−0.0131 to 0.0495
Proposed vs. Without spatial filters	Balanced Accuracy	0.8802	0.8254	+0.0548	−0.0055 to 0.1212
	Sensitivity	0.8250	0.6750	**+0.1500**	**0.0333 to 0.2745**
	Specificity	0.9355	0.9758	**−0.0403**	**−0.0840 to −0.0079**
	AUROC	0.9522	0.9677	**−0.0155**	**−0.0337 to −0.0002**

Bold values represent the metric values obtained using patient level clustered bootstrap of the proposed model.

In addition, differences in AUROC were evaluated using paired DeLong tests with Holm adjustment. Because multiple test observations originated from the same patient, patient-level clustered bootstrap resampling with 5,000 replicates was also performed. Patients rather than individual observations were resampled with replacement, and all observations belonging to each selected patient were retained. This analysis provided 95% confidence intervals for paired differences in balanced accuracy, sensitivity, specificity, F1-score, MCC, AUROC, and average precision.

Cochran’s Q and exact McNemar analyzes did not identify statistically significant differences in overall paired classification correctness among the component-ablation models after multiple-comparison correction. Similarly, the paired DeLong tests did not demonstrate significant AUROC differences after Holm correction. Patient-level clustered bootstrap analysis nevertheless revealed several operating-point trends. In particular, removal of the spatial-filtering branches reduced malignant-case sensitivity, while the model without spatial filters exhibited greater specificity and AUROC. These findings indicate a sensitivity-specificity trade-off rather than uniform superiority of one configuration across all evaluation metrics.

#### 5.3.5 Overall ablation findings

The ablation experiments support three observations, each stated here at the strength the statistical analysis actually supports. First, the distribution of the Lag-Lead compensator across several feature-extraction stages yielded numerically satisfactory performance as compared to the insertion of the compensator in a single stage, although Cochran’s Q testing of the nine stage-wise configurations was not statistically significant (*p* = 0.1579; Table 15), so this is best characterized as a consistent numerical trend rather than a confirmed effect. Second, the model performed competitively on the class-imbalance ratios investigated, which suggests the effectiveness of the model is not limited to a single benign-malignant ratio. Third, the combined Lag-Lead configuration achieved the best sensitivity and balanced accuracy compared to the Lag-only, Lead-only, and combined branch configurations (5.3.5). However, the corresponding AUROC differences were not statistically significant after Holm correction (Table 16), so the combined configuration is best described as providing the most favorable operating-point trade-off rather than consistently outperforming the individual branches. These results are in line with the architectural design of the proposed model, where the final performance is the joint contribution of multistage insertion and combined Lag-Lead feature compensation, but they should be read as supporting report for a design choice rather than statistically confirmed proof of superiority.

**Table 15:** Cochran’s Q and exact McNemar statistical analysis of stage-wise configurations.

Analysis	No. of models	Test cases	Statistic	df	*p*-value	No. of pairwise tests	Significant before correction	Significant after Holm
Cochran’s Q + exact McNemar	9	164	11.854	8	0.1579	8	0	0

**Table 16:** DeLong analysis of AUROC differences across component ablations.

Comparison	Full AUROC	Comparison AUROC	ΔAUROC (full − other)	DeLong Z	Raw *p*-value	Holm-adjusted p	Significant
Full proposed vs. Lag only	0.9522	0.9423	+0.0099	0.7114	0.4768	0.6965	No
Full proposed vs. Lead only	0.9522	0.9361	+0.0161	0.9380	0.3483	0.6965	No
Full proposed vs. Without spatial filters	0.9522	0.9677	−0.0155	−1.9514	0.0510	0.1530	No

#### 5.3.6 Influence of Lag-Lead compensations in proposed work

The distinct roles of the Lag and Lead component are revealed by component-ablation analysis. On the same held-out patient level test, the Lag-only configuration achieved an accuracy of 0.92, balanced accuracy of 0.90, sensitivity of 0.85, specificity of 0.94, F1-score of 0.84, MCC of 0.78, and AUROC of 0.94. The results suggest that the Lag compensation alone retains useful discriminative information and maintains relatively balanced specificity for malignant cases and detection of benign cases.

The Lead-only configuration showed a slightly better accuracy (0.92), specificity (0.97), F1-score (0.83) and MCC (0.79). But its sensitivity was down to 0.77. This trend indicates that targeting boundary- and high-frequency related information leads to more robust benign-case rejection but may shift the operating point toward higher specificity at the expense of malignant-case sensitivity.

The full Lag-Lead setup presented a different trade-off. It reached an accuracy of 0.90, balanced accuracy of 0.90, sensitivity of 0.90, specificity of 0.90, F1-score of 0.81, and AUROC of 0.95. The complete model thus gave the best malignant-case sensitivity and best balanced accuracy among the Lag-only, Lead-only and combined set-ups, although Lag-only and Lead-only gave higher overall accuracy and specificity, respectively.

This behavior is further explained by the ablation without spatial filtering. When the spatial-filtering mechanisms were removed, accuracy was 0.90 with high specificity of 0.97, but sensitivity was reduced to 0.67. patient-level bootstrap analysis confirmed an absolute sensitivity difference of 0.15 (95%CI:0.0333–0.2745). Interestingly, this setting achieved a higher AUROC of 0.96, indicating that the threshold-independent ranking ability and the operating-point malignant sensitivity do not always move in the same direction. Collectively, these results demonstrate that the Lag and Lead compensations are not equivalent. The Lead compensation is biased toward specificity and the combined compensation is more sensitive to malignant samples. The spatial-filtering mechanism thus appears to affect the sensitivity-specificity operating trade-off rather than to uniformly boost all performance measures. This interpretation is also consistent with the statistical analysis: the component-level differences observed were not statistically significant after Holm correction and thus should be interpreted as performance trends rather than statistically confirmed superiority.

### 5.4 External validation image-level dataset

The External dataset used in this work was the IQ-OTH/NCCD (Al-Yasriy, 2023) lung cancer CT dataset obtained from Kaggle. The original public dataset contains CT images from normal, benign, and malignant lung cancer categories, with an image-level distribution of 120 benign images, 561 malignant images, and 416 normal images. Since the objective of this work was binary benign vs. malignant lung nodule classification, the normal category was excluded from the experimental analysis. Although the original dataset contains 561 malignant images, this work used an experimental subset consisting of 120 benign images and 561 malignant images. To reduce evaluation bias, stratified train, validation, and test partitioning was applied so that both benign and malignant classes were represented in each split. The class imbalance was considered during model training and evaluation, and the model performance was reported using multiple metrics, including accuracy, precision, recall, F1-score, and confusion matrix analysis, rather than accuracy alone.

The public IQ-OTH/NCCD release does not provide reliable volumetric patient identifiers for strict patient-level partitioning. Therefore, the dataset was divided at the image-slice level, and this is acknowledged as a methodological limitation. The dataset was partitioned using 5-fold cross-validation at the image level to preserve class balance across splits. Because reliable patient identifiers are not available in the public release, slice level leakage between folds cannot be excluded, and this dataset is accordingly reported throughout as an exploratory, image-level evaluation rather than a patient-level external validation.

For each fold, the data were divided into training (~70%), validation (~15%), and test (~15%) subsets, with class proportions (benign: malignant) maintained approximately constant across all three subsets in every fold. Table 17 summarizes the per-fold sample counts. Minor variation in absolute counts across folds (e.g., 415–416 training samples) reflects the indivisibility of the total sample count into exactly equal folds; class ratios were held stable via stratified sampling. Augmentation was applied only to training images after partitioning. Because slice-level rather than patient-level separation was used, the results reported for this dataset should be interpreted with more caution than the patient-level LIDC-IDRI and BUS-BRA results reported elsewhere in this work and are presented as a supplementary, exploratory check on cross-dataset behavior rather than as confirmatory external validation.

**Table 17:** IQ-OTH (image-level) dataset distribution used in this work.

Fold	Total train images	Train Benign/Malignant	Total validation images	Validation Benign/Malignant	Test total	Test Benign/Malignant
1	415	66/349	104	16/88	130	20/110
2	415	66/349	104	16/88	130	20/110
3	415	66/349	104	16/88	130	20/110
4	415	66/349	104	16/88	130	20/110
5	415	66/349	104	16/88	129	19/110

For the IQ-OTH Lung Cancer dataset in Table 17, a 5-fold experimental protocol was adopted for evaluation. The training, validation and held-out test subsets were kept separate within each fold. The training set consisted of about 415 images, the validation set had 104 images and the testing set contained 129–130 images per fold. The distribution of classes in each fold is summarized in Table 17.

The proposed model achieved good performance on the IQ-OTH Lung Cancer dataset, with an accuracy of 0.98, precision of 0.99, recall of 0.98, F1-score of 0.99, AUROC of 0.99, and MCC of 0.95 as presented in Tables 18, 19. Results show that the numerical discrimination under the image-level five-fold evaluation protocol.

**Table 18:** Performance of the proposed model across independent IQ-OTH (image-level) datasets.

Datasets	Accuracy	Precision	Recall	F1-score	AUROC	MCC
IQ-OTH lung cancer (5-FOLD RESULTS)	0.98	0.99	0.98	0.99	0.99	0.95

**Table 19:** Performance of the ROC, PR-AUC curves of proposed model across independent IQ-OTH (image-level) datasets.

IQ-OTH Lung Cancer (5-FOLD RESULTS)	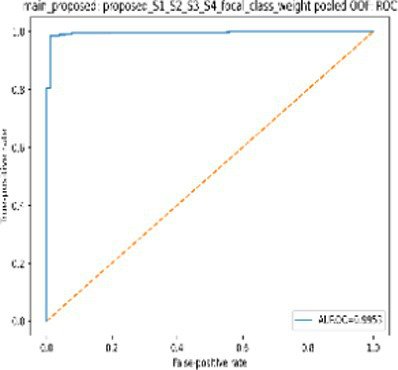	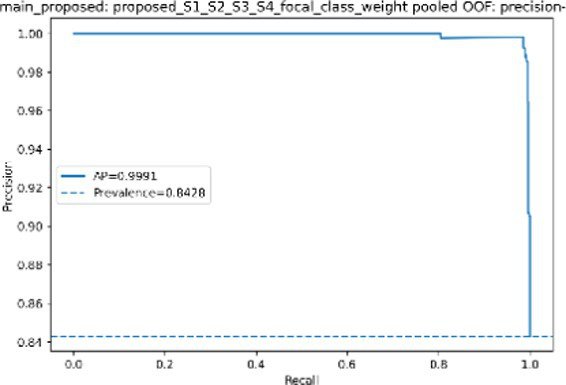	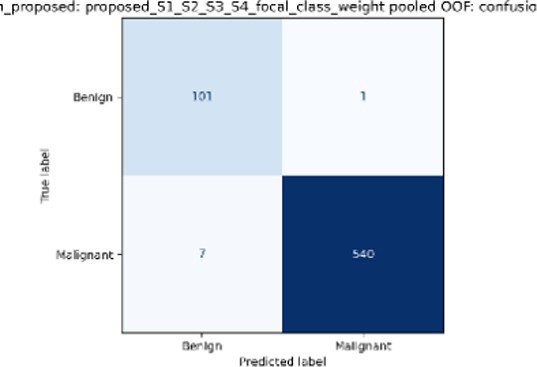

While these values were lower than those obtained on IQ-OTH, the AUROC of 0.89 shows that the framework was able to discriminate useful information in a substantially different data distribution. The lower recall indicates that positive cases were harder to find in this dataset, demonstrating the importance of dataset characteristics and domain differences on operating-point performance.

### 5.5 Generalization analysis

For binary breast lesion classification, an extra benchmark, the BUS-BRA (Gómez-Flores et al., 2024) (Breast Ultrasound-Brazil) dataset, was used. The dataset includes 1,875 anonymized breast ultrasound images obtained from 1,064 female patients undergoing routine breast examinations at the National Institute of Cancer, Rio de Janeiro, Brazil. The images were acquired with four different ultrasound scanners, thus introducing realistic variations of image acquisition characteristics.

All tumors in the dataset are confirmed by biopsy and classified as benign or malignant. The dataset has 722 benign patients and 342 malignant patients at the patient level which corresponds to 1,268 benign images and 607 malignant images at the patient level. Apart from the diagnostic labels, the BUS-BRA dataset also contains BI-RADS assessments in the range of 2 to 5 and manually delineated lesion boundaries that can be used as ground-truth annotations.

During data preparation, patient identifiers were maintained to prevent information leakage across training and evaluation subsets, as multiple ultrasound images could belong to the same patient. None of the patients in the processed dataset used in this work had mixed benign and malignant image labels, therefore each patient could only be assigned to one diagnostic class unambiguously. Therefore, patient-level grouping can be applied during the dataset partitioning so that all images of a given patient are only present in the same training, validation or testing subset.^2^

#### 5.5.1 Independent-dataset evaluation

To investigate the robustness of the proposed framework beyond the primary dataset, we conducted additional experiments on two independent datasets with distinct data distribution and evaluation protocols, namely, the IQ-OTH Lung Cancer dataset and the BUS-BRA dataset. The purpose of these experiments is to test whether the proposed architecture can still discriminate effectively on datasets with different sample characteristics and class distributions.

For BUS-BRA in Table 20, we used patient-level partitioning to reduce the information leakage between the training, validation and test sets. The dataset consisted of 1,875 images from 1,064 patients. 1,313 images of 744 patients were used for training, and 281 images of 160 patients were used for validation and 281 images of 160 patients were used for testing. Table 20 summarizes the corresponding class distributions.

**Table 20:** BUS-BRA (patient-level) dataset distribution used in this work.

Split	Images	Patients	Benign_images	Malignant_images
Train	1,313	744	888	425
Validation	281	160	190	91
Test	281	160	190	91
Total	1875	1,064	1,268	607

The model achieved 0.84 accuracy, 0.80 precision, 0.67 recall, 0.73 F1-score, 0.89 AUROC and 0.62 MCC on the BUS-BRA dataset as shown in Table 20 and its corresponding results shown in Table 21.

**Table 21:** Performance of the proposed model across independent datasets.

Datasets	Accuracy	Precision	Recall	F1-score	AUROC	MCC
BUS-BRA (patient-level)	0.84	0.80	0.67	0.73	0.89	0.62

In conclusion, the independent-dataset experiments show that the proposed architecture can sustain useful classification performance for datasets with different characteristics. But the difference in the performance especially on the BUS-BRA indicates the dataset dependency of the models. So we take these results (Table 22) as a sign of robustness across datasets rather than generalizability.

**Table 22:** Performance curves of the proposed model across independent datasets.

BUS-BRA (patient-level)	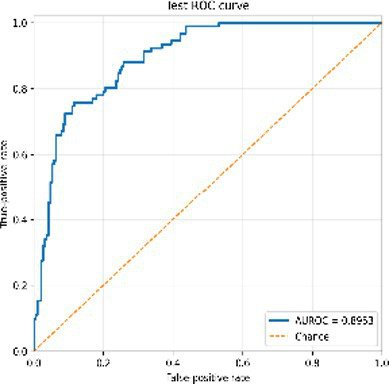	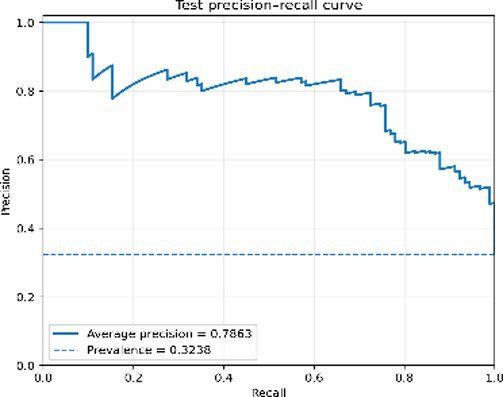	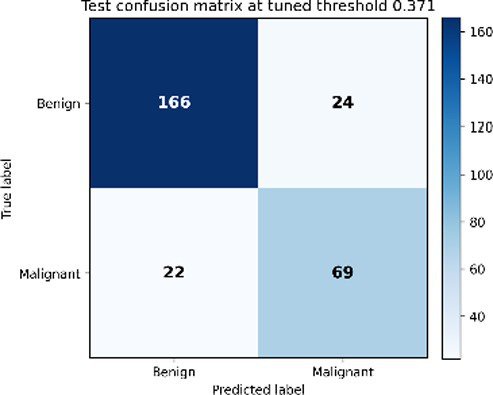

### 5.6 Statistical significance analysis

Statistical comparison was performed at both the classification-correctness and discrimination levels. First, pairwise differences between two model configurations were evaluated using the exact McNemar test, followed by the Holm correction to account for multiple pairwise comparisons. Next, Cochran’s Q test was applied as an omnibus paired test to determine whether the probability of correct classification differed across multiple model configurations. Since McNemar’s and Cochran’s Q tests are based on binary correct/incorrect outcomes at the selected decision threshold, paired AUROC differences were assessed separately using DeLong’s test, with Holm adjustment for multiple comparisons. Thus, McNemar’s test evaluated pairwise disagreement in classification outcomes, Cochran’s Q test assessed overall differences among multiple classifiers, and DeLong’s test examined threshold-independent differences in discriminative performance.

Because all model configurations were evaluated on the same held-out test samples, paired statistical procedures were used to assess whether the observed performance differences were statistically supported. For comparisons involving multiple related classifiers, Cochran’s Q test was employed as an omnibus non-parametric test based on sample-wise classification correctness. Each model prediction was represented as a binary outcome, with 1 denoting correct classification and 0 denoting incorrect classification. The null hypothesis assumes an equal probability of correct classification across all evaluated models.

For pairwise comparisons of classification correctness, the exact McNemar test was used. The test considers only discordant observations, i.e., cases correctly classified by one model but incorrectly classified by the other. Because some comparisons contained relatively few discordant cases, the exact binomial form of McNemar’s test was adopted. To account for multiple pairwise comparisons, *p*-values were adjusted using the Holm procedure, with statistical significance defined as an adjusted. For the nine stage-wise configurations, Cochran’s Q test produced Q = 11.854, df = 8, *p* = 0.158 as shown in Table 15.

### 5.7 AUROC comparison

Because Cochran’s Q (1950) and McNemar’s (1947) tests evaluate binary classification correctness rather than discrimination across all possible thresholds, differences in AUROC were separately assessed using the paired DeLong test. The Full Proposed model was compared with the Lag-only, Lead-only, and Without Spatial-Filters configurations. Holm adjustment was again applied to control for multiple comparisons.

The Full Proposed model achieved an AUROC of 0.9522, compared with 0.9423 for the Lag-only model and 0.9361 for the Lead-only model. The corresponding AUROC differences were and, respectively; however, neither comparison was statistically significant after Holm correction (Holm, 1979) (for both comparisons).

The model without spatial filters achieved a numerically higher AUROC of 0.9677, corresponding to a Full-minus-comparison difference of −0.0155. The unadjusted DeLong (1988) test approached the conventional significance threshold (0.05), but the difference was not statistically significant after Holm correction (0.15). Consequently, the AUROC results do not establish statistically significant superiority of either the Full Proposed model or any of the component-ablation variants as shown in Table 16.

Thus, no statistically significant overall difference in classification correctness was detected among the nine configurations. Eight planned pairwise McNemar comparisons were subsequently examined; none reached statistical significance either before or after Holm correction. Therefore, although numerical differences in accuracy were observed among the stage configurations, the paired classification outcomes did not provide sufficient results to establish statistically significant superiority of any individual configuration on the evaluated test set.

Overall, the statistical analyzes indicate that the numerical performance differences observed among the proposed, stage-wise, and component-ablation configurations were not statistically significant after correction for multiple comparisons. Cochran’s Q and exact McNemar tests showed no significant differences in paired classification correctness, while DeLong analysis similarly showed no significant pairwise AUROC differences. These findings suggest that the reported performance variations should be interpreted as numerical trends rather than statistically confirmed superiority. Nevertheless, the ablation results remain informative for characterizing the distinct operating behavior of the Lag, Lead, and spatial-filtering components, particularly with respect to sensitivity, specificity, and threshold-independent discrimination.

## 6 Conclusion

This proposed work Bilinear Lag-Lead, a frequency response compensation framework that embeds bilinear-transform derived Lag and Lead filters within EfficientNetB3, fused through an efficient learnable projection at selected stages. Under patient-level held-out evaluation on LIDC-IDRI, the complete four-stage configuration achieved an accuracy of 0.90, balanced accuracy of 0.90, sensitivity of 0.90, specificity of 0.90, F1-score of 0.81, and AUROC of 0.95. Performance was further characterized through parameter-sensitivity analysis, class-imbalance testing, an exploratory image-level external evalution and a patient-level cross-dataset robustness assessment. Component ablation showed that the Lag and Lead exhibit distinct response characteristics and their combination achieved the best sensitivity and balanced accuracy among branch-based configurations. Patient-level clustered bootstrap analysis demonstrated that the proposed frequency-based spatial compensation provides a measurable advantage in malignant-case detection. In particular, removing the spatial filters reduced sensitivity from 0.8250 to 0.6750, corresponding to an absolute improvement of 0.1500 for the proposed model (95% CI: 0.0333–0.2745). This statistically supported gain confirms the functional contribution of the Lag-Lead compensation mechanism, while the accompanying changes in specificity and AUROC indicate a sensitivity-oriented operating trade-off. Accordingly, the present work should be regarded as a methodological investigation providing a reproducible foundation for subsequent external and prospective validation, rather than a clinically deployment-ready diagnostic system.

### 6.1 Limitations

The proposed work was evaluated using patient-level data separation and multiple statistical tests on the primary LIDC-IDRI and BUS-BRA datasets, with the exception of the IQ-OTH/NCCD evaluation, which used image level partitioning because reliable patient identifiers were not available in the public release and is accordingly reported as an exploratory rather than confirmatory validation. However, several limitations remain. The relatively small held-out cohort and reliance on radiologist-assessed malignancy labels rather than uniformly pathology-confirmed diagnoses limit the strength of clinical generalization. In addition, the frequency-response compensation cannot be assumed to be invariant to acquisition-related domain shifts, since variations in scanner manufacturer, radiation dose, slice thickness, reconstruction kernel, spatial resolution, and preprocessing may alter CT frequency characteristics and affect model performance. Therefore, the present findings should be regarded as preliminary reports, and future work should include larger pathology-confirmed external cohorts, multicenter validation, acquisition-stratified analysis, image-quality perturbation testing, and calibration assessment across different CT protocols.

### 6.2 Future work

- External multi-center validation: Evaluate the model on larger independent cohorts acquired using different scanners, reconstruction settings, slice thicknesses, and institutional protocols to assess robustness to real-world domain variation.
- Extension to 2.5D/3D analysis: Develop volumetric BT-LL models so that spatial-frequency compensation can exploit complete nodule morphology across adjacent CT slices rather than relying only on 2D representations.
- Pathology-confirmed validation: Evaluate the framework on cohorts with histopathological ground truth to distinguish prediction of radiologist-assessed malignancy from prediction of clinically confirmed malignancy.

## Data Availability

The original contributions presented in the study are included in the article/Supplementary material, further inquiries can be directed to the corresponding author.

## Footnotes

1
https://www.cancerimagingarchive.net/collection/lidc-idri/

2
https://zenodo.org/records/8231412
