# Supplementary material for: Frequency response compensation to optimize pretrained deep learning models in lung nodule malignancy classification
Source: Front Artif Intell. 2026 Sep 22;9:1934912. doi: 10.3389/frai.2026.1934912 (PMC13638547; doi:10.3389/frai.2026.1934912)
Supplement: Supplementary file 1 [file Data_Sheet_1.DOCX]

Supplementary Material

# 1 Supplementary Data

(<https://www.kaggle.com/datasets/hamdallak/the-iqothnccd-lung-cancer-dataset>

<https://www.kaggle.com/datasets/raddar/nodules-in-chest-xrays-jsrt?select=images>

<https://github.com/sangeethabala83/-Frequency-Response-Compensation.git>

# 2 Supplementary Tables

**Table 1. Original IQ-OTH/NCCD dataset and experimental subset used in this work**

| **Category** | **Original dataset count** | **Used in this study** | **Excluded** | **Reason** |
| --- | --- | --- | --- | --- |
| Normal | 416 | 0 | 416 | Excluded because this work focused on benign vs malignant classification |
| Benign | 120 | 120 | 0 | All available benign images were used |
| Malignant | 561 | 350 | 211 | Random malignant subset selected before training |
| **Total** | **1097** | **470** | **627** | Final experimental subset used for binary classification |

**Table 2 Classification Performance of the Proposed Model**

|  |  |  |
| --- | --- | --- |

**Table 3 Ablation study results**

| **Model Name** | **Precision** | **Recall** | **F1-Score** | **Accuracy** |
| --- | --- | --- | --- | --- |
| EfficientNetB3 | 0.86 | 0.87 | 0.84 | 0.88 |
| Lag Compensation | 0.51 | 1.00 | 0.68 | 0.53 |
| Lead Compensation | 0.90 | 1.00 | 0.89 | 0.94 |
| **Proposed Model** | **0.95** | **1.00** | **0.97** | **0.97** |

**Table 4 Parameter Sensitivity Analysis**

|  |  |  |
| --- | --- | --- |
|  |  |  |
|  |  |  |
|  |  |  |
|  |  |  |
|  |  |  |
|  |  |  |
|  |  |  |
|  |  |  |
|  |  |  |
|  |  |  |
|  |  |  |
|  |  |  |
|  |  |  |
|  |  |  |
|  |  |  |
|  |  |  |
|  |  |  |
|  |  |  |
|  |  |  |
|  |  |  |
|  |  |  |
|  |  |  |
|  |  |  |
|  |  |  |
|  |  |  |
|  |  |  |

**Table 5 Parameter sensitivity results for different** $\boldsymbol{T}_{\boldsymbol{1}}$**and** $\boldsymbol{T}_{\boldsymbol{2}}$**combinations**

| **(T1)** | **(T2)** | **Accuracy** | **Precision** | **Recall** | **F1-score** |
| --- | --- | --- | --- | --- | --- |
| 0.1 | 0.05 | 1.0000 ± 0.0000 | 1.0000 ± 0.0000 | 1.0000 ± 0.0000 | 1.0000 ± 0.0000 |
| 0.1 | 0.10 | 0.9872 ± 0.0222 | 0.9762 ± 0.0412 | 1.0000 ± 0.0000 | 0.9877 ± 0.0214 |
| 0.1 | 0.15 | 0.9936 ± 0.0111 | 0.9877 ± 0.0214 | 1.0000 ± 0.0000 | 0.9937 ± 0.0109 |
| 0.2 | 0.05 | 1.0000 ± 0.0000 | 1.0000 ± 0.0000 | 1.0000 ± 0.0000 | 1.0000 ± 0.0000 |
| 0.2 | 0.10 | 1.0000 ± 0.0000 | 1.0000 ± 0.0000 | 1.0000 ± 0.0000 | 1.0000 ± 0.0000 |
| 0.2 | 0.15 | 0.9936 ± 0.0111 | 0.9877 ± 0.0214 | 1.0000 ± 0.0000 | 0.9937 ± 0.0109 |
| 0.3 | 0.05 | 1.0000 ± 0.0000 | 1.0000 ± 0.0000 | 1.0000 ± 0.0000 | 1.0000 ± 0.0000 |
| 0.3 | 0.10 | 1.0000 ± 0.0000 | 1.0000 ± 0.0000 | 1.0000 ± 0.0000 | 1.0000 ± 0.0000 |
| 0.3 | 0.15 | 1.0000 ± 0.0000 | 1.0000 ± 0.0000 | 1.0000 ± 0.0000 | 1.0000 ± 0.0000 |

**Table 6 Feature Map Visualization of before and After Lag-Lead**

|  |  |  |  |
| --- | --- | --- | --- |
|  |  |  |  |

**Table 7. Original dataset distribution used in this work**

| **Dataset** | **Modality** | **Original Benign/Malignant** | **Original Total** | **Selected**  **Benign/Malignant** | **Experimental Total** | **Excluded** |
| --- | --- | --- | --- | --- | --- | --- |
| **IQ-OTHNCCD** | CT | 120/561 | 681 | 120/350 | 470 | 211 |
| **JSRT lung-nodule dataset** | X-ray | 54/100 | 154 | 54/54 | 108 | 46 |
| **BreakHis** | Histopathology | 2480/5429 | 7909 | 1000/1000 | 2000 | 5909 |

**Table 8. Performance of the proposed model across independent datasets**

| **Dataset** | **Modality** | **Accuracy** | **Recall** | **Precision** | **F1-Score** |
| --- | --- | --- | --- | --- | --- |
| **IQ-OTHNCCD** | CT | 0.97 | 1.00 | 0.95 | 0.97 |
| **X-ray Lung Nodule (JSRT)** | X-ray | 0.66 | 1.00 | 0.66 | 0.80 |
| **BreakHis** | Histopathology | 0.95 | 0.89 | 1.00 | 0.94 |

**Table 9 Comparative Analysis of EfficientNet Variance Models**

| **Methods** | **Precision** | **Recall** | **F1-score** | **Accuracy** |
| --- | --- | --- | --- | --- |
| EfficientNetB0 | 0.90 | 0.90 | 0.89 | 0.90 |
| EfficientNetB1 | 0.93 | 0.93 | 0.92 | 0.93 |
| EfficientNetB2 (34) | 0.94 | 0.96 | 0.91 | 0.96 |
| EfficientNetB3 | 0.93 | 0.93 | 0.93 | 0.93 |
| EfficientNetB4 | 0.93 | 0.78 | 0.85 | 0.86 |
| EfficientNetB5 | 0.98 | 0.97 | 0.97 | 0.97 |
| EfficientNetB6 | 1.00 | 0.88 | 0.94 | 0.94 |
| EfficientNetB7 | 0.99 | 0.91 | 0.95 | 0.95 |
| **Proposed Model** | **0.95** | **1.00** | **0.97** | **0.97** |

**Table 10 Comparative Analysis of Different Models**

| **Methods** | **Precision** | **Recall** | **F1-score** | **Accuracy** |
| --- | --- | --- | --- | --- |
| SE-ViT (38) | 0.87 | 0.87 | 0.87 | 0.86 |
| V-Net (35) | 0.91 | 0.86 | 0.88 | 0.91 |
| Ensemble CNN (36) | 0.97 | 0.93 | 0.93 | 0.96 |
| SSTL-FM | 0.94 | 0.92 | 0.93 | 0.93 |
| Spinal-EfficientNet (32) | 0.92 | 0.92 | 0.91 | 0.92 |
| **Proposed Model** | **0.95** | **1.00** | **0.97** | **0.97** |

**Table 11 Performance with Wilson 95% confidence intervals**

| **Model** | **Accuracy (95% CI)** | **Precision (95% CI)** | **Recall (95% CI)** | **Specificity (95% CI)** |
| --- | --- | --- | --- | --- |
| Lag compensation | 0.528 (0.370–0.680) | 0.514 (0.356–0.670) | 1.000 (0.824–1.000) | 0.056 (0.010–0.258) |
| Lead compensation | 0.944 (0.819–0.985) | 0.900 (0.699–0.972) | 1.000 (0.824–1.000) | 0.889 (0.672–0.969) |
| Proposed model | 0.972 (0.858–0.995) | 0.947 (0.754–0.991) | 1.000 (0.824–1.000) | 0.944 (0.742–0.990) |
